# A Scaled Proteomic Discovery Study for Prostate Cancer Diagnostic Markers Using Proteograph^TM^ and Trapped Ion Mobility Mass Spectrometry

**DOI:** 10.3390/ijms25158010

**Published:** 2024-07-23

**Authors:** Matthew E. K. Chang, Jane Lange, Jessie May Cartier, Travis W. Moore, Sophia M. Soriano, Brenna Albracht, Michael Krawitzky, Harendra Guturu, Amir Alavi, Alexey Stukalov, Xiaoyuan Zhou, Eltaher M. Elgierari, Jessica Chu, Ryan Benz, Juan C. Cuevas, Shadi Ferdosi, Daniel Hornburg, Omid Farokhzad, Asim Siddiqui, Serafim Batzoglou, Robin J. Leach, Michael A. Liss, Ryan P. Kopp, Mark R. Flory

**Affiliations:** 1 Cancer Early Detection Advanced Research Center, Knight Cancer Institute, Oregon Health and Science University, Portland, OR 97201, USA; changma@ohsu.edu (M.E.K.C.); ssoriano@sumbeach.com (S.M.S.);; 2 Department of Cell Systems and Anatomy, University of Texas Health San Antonio, San Antonio, TX 78229, USA; 3 Bruker Daltonics, Billerica, MA 01821, USA; 4 Seer Inc., Redwood City, CA 94065, USA; 5 Roger L. & Laura D. Zeller Charitable Foundation in Urologic Oncology, University of Texas Health San Antonio, San Antonio, TX 78229, USA

**Keywords:** prostate cancer, biomarker, serum, proteomics, mass spectrometry, diagnosis

## Abstract

There is a significant unmet need for clinical reflex tests that increase the specificity of prostate-specific antigen blood testing, the longstanding but imperfect tool for prostate cancer diagnosis. Towards this endpoint, we present the results from a discovery study that identifies new prostate-specific antigen reflex markers in a large-scale patient serum cohort using differentiating technologies for deep proteomic interrogation. We detect known prostate cancer blood markers as well as novel candidates. Through bioinformatic pathway enrichment and network analysis, we reveal associations of differentially abundant proteins with cytoskeletal, metabolic, and ribosomal activities, all of which have been previously associated with prostate cancer progression. Additionally, optimized machine learning classifier analysis reveals proteomic signatures capable of detecting the disease prior to biopsy, performing on par with an accepted clinical risk calculator benchmark.

## 1. Introduction

Prostate cancer is the most commonly diagnosed non-cutaneous cancer and the second leading cause of cancer-related death among U.S. men [1]. For decades, the clinical measurement of the prostate-specific antigen (PSA) protein in blood has been the primary method for detecting prostate cancer. Since the advent of PSA screening in the early 1990s, prostate cancer mortality in the U.S. has decreased by almost 50% [2,3,4], and today PSA remains the principal marker used for detection, progression, and, in certain contexts, predicting the clinical outcomes of prostate cancer [5,6]. 

However, PSA screening is controversial due to its association with several harms, including the overdiagnosis and overtreatment of indolent disease and unnecessary biopsies in men with elevated PSA but no cancer [7,8,9,10]. In support of this concern, for a PSA cutoff of 4.0 ng/mL, a significant percentage of prostate biopsies result in negative findings, indicating that many of these biopsies were unnecessary [11]. Despite advancements in diagnostic strategies that combine PSA blood testing and prostate imaging through MRI (magnetic resonance imaging) for detection prior to biopsy, negative biopsies occur in roughly 30–40% cases [12,13,14,15]. While existing follow-on tests contingent on elevated PSA, so-called PSA reflex tests [16,17], and multiparametric MRI [18] reduce at least a fraction of unnecessary biopsies in men with PSA levels of 4–10 ng/mL, there is still a significant unmet clinical need for improved reflex tests capable of more accurately discriminating prostate cancer’s presence from other causes of elevated PSA while minimizing false negatives. Ideally, such tests would be less costly, less time-consuming, less resource-intensive, and more practical for patients compared to prostate MRI. Ultimately, such improved tests would provide more informed decisions regarding the necessity of an invasive prostate biopsy.

While various approaches are being explored to identify biomarkers for new PSA reflex tests [19], proteomics is an especially attractive approach for this indication given that methylated DNA signatures in blood, though highly predictive for other cancers, show relatively low sensitivity for the detection of prostate cancer [20]. For true discovery-mode proteomics facilitating new biomarker discovery, liquid chromatography-coupled tandem mass spectrometry (LC-MS) remains the most powerful approach. However, sample preparation methods upstream of LC-MS have historically failed to overcome the enormous dynamic range of protein abundances in this biofluid, precluding feasible, deep, and scalable proteomic sampling necessary for effective biomarker discovery [21]. The Seer Proteograph^TM^ workflow employed here overcomes this barrier by significantly enhancing the depth, consistency, and scalability of serum and plasma proteomic profiling compared to traditional methods that rely on abundant protein depletion and sample fractionation. The platform uses proprietary engineered nanoparticles (NPs) to selectively capture proteins from neat plasma or serum across the entire dynamic range [22], and the technical and scientific underpinnings of this solution have been extensively studied. The automation of this platform enables interrogation of large specimen cohorts, helps to overcome inter-patient sample variance, and provides sufficiently large output datasets necessary for machine learning strategies to detect subtle signals across multiple features. The initial publication describing Proteograph [22] presented the novel use of nanoparticles to improve the depth of proteins quantified, providing a 5x boost in identifications over a depletion workflow. Ferdosi et al., 2022 presented a Proteograph workflow with superior depth and coefficients of variation versus fractionation and depletion workflows [23], and additional work provided insights into how the nanoparticles function at a mechanistic level [24]. Proteograph has since been shown to aid in the discovery of cancer-related isoforms [25], biological alterations induced by spaceflight [26,27], and changes in biological pathways of muscle tissue in response to exercise [28]. Proteograph has also been demonstrated to facilitate cohort proteogenomics by providing peptide-level information, allowing for resolution of MS artifacts in protein quantitative trait loci (pQTL) identification [29], with a recent follow-up preprint publication [30] claiming to identify potentially false pQTLs in affinity-based methods.

We combine Proteograph with the robust and high-sensitivity Bruker timsTOF (trapped ion mobility time-of-flight) ion mobility mass spectrometry (MS) [31,32] to identify new diagnostic markers within a large cohort of prostate cancer patients’ serum. To maximize proteomic coverage and data completeness, we employ a specialized form of data-independent acquisition (DIA) on the timsTOF (dia-PASEF, DIA parallel accumulation serial fragmentation) [33] and further augment our datasets with a study-specific spectral library. We implement a rigorous study design crafted to avoid common pitfalls of biomarker discovery, such as failure to define a clear clinical endpoint, insufficient interdisciplinary expertise, or lack of rigor in study design and poor selection of cases and controls, all factors that reduce the potential for discovered signatures to effectively translate to clinical assays [34]. We hypothesize that a combination of these two proteomic platforms in the context of a rigorous, clinically well-defined study design will significantly increase our ability to uncover proteomic blood signatures for downstream PSA reflex tests, thus improving prostate cancer diagnosis.

Our discovery campaign for new PSA reflex markers achieves remarkable proteomic depth in a large-scale patient serum cohort. Differential protein abundance analysis across the study groups reveals a significant number of previously reported prostate cancer blood markers and a rich set of novel candidate blood markers, including those with ascribed regulatory function in prostate cancer progression. Machine-learning-based analysis reveals new proteomic signatures that achieve a disease classifier performance near that of a current clinical diagnostic tool, the Prostate Cancer Prevention Trial (PCPT) risk calculator version 1.0 [35]. The research marks an early step in the inevitably extensive road towards new clinical test deployment, providing valuable new proteomic biomarker candidates in blood for future validation in expanded and diverse patient cohorts.

## 2. Results

### 2.1. Study Population and Design

**Figure 1:**
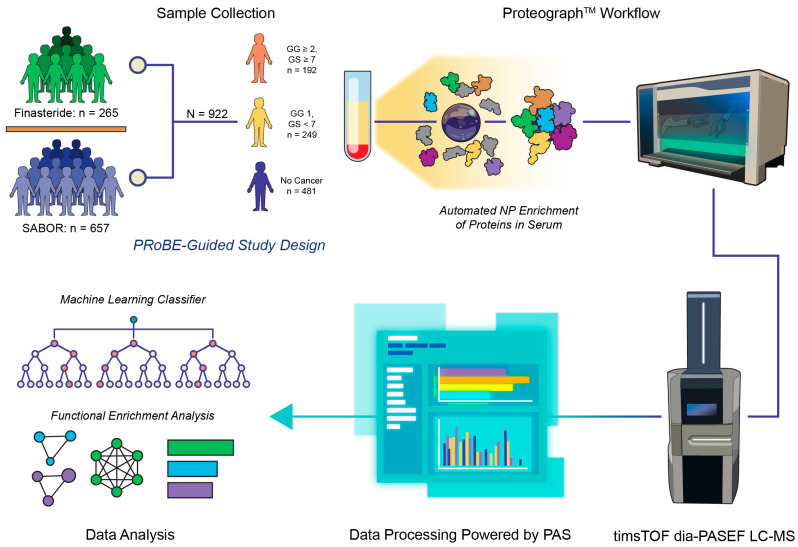
Study schematic. Design of the study was rooted in the principles of biomarker discovery as described by PRoBE (prospective specimen collection, retrospective blinded evaluation). Subjects were included from two different cohorts for prostate cancer, Finasteride and San Antonio Center of Biomarkers for Risk for Prostate Cancer (SABOR), where serum was collected according to the Early Detection Research Network (EDRN) protocol. Patients referred for biopsy due to elevated PSA were divided into three categories (Gleason Grade Group GG ≥ 2, GG 1, and no cancer) based on the results of prostatectomy or biopsy. Serum was prepared for proteomic analysis using the Seer Proteograph^TM^ nanoparticle (NP) protein enrichment workflow, and resulting peptides were analyzed through dia-PASEF LC-MS. Raw DIA MS data were processed with DIA-NN in the Seer cloud-based Proteograph Analysis Suite (PAS). Subsequent output data were used to train a random forest-based multi-marker cancer classifier and analyzed using functional enrichment for known biological pathways.

To execute a biomarker discovery study best served for clinical impact, we utilized the PRoBE framework to create a rigorous study design and to choose a set of subjects emblematic of this study’s targeted, downstream prostate cancer test’s endpoint population. To this end, we utilized banked serum that was collected using a consistent Early Detection Research Network (EDRN) protocol [36] from N = 922 subjects enrolled in two cohorts, the Finasteride Study Challenge (FSC) [37] and the San Antonio Center of Biomarkers for Risk for Prostate Cancer (SABOR) [38]. All men in the study were referred for prostate biopsy due to elevated PSA or abnormal digital rectal exam (DRE), with the resultant prostate cancer status determined through biopsy. We utilized grade on prostatectomy for a subset who experienced upgrading or downgrading at surgery occurring within 18 months of the initial blood draw. In the final study population, patients diagnosed with prostate cancer (n = 441) were further divided into clinically significant cancer (Gleason Grade Group GG ≥ 2) and low-grade cancer (GG 1) compared to n = 481 subjects with no cancer (Figure 1). Additional information on the patient population and specific recruitment criteria are detailed in Section 4.1 and Section 4.2 and summarized in Table 1. Samples were divided into a training (n = 642) and validation (n = 280) set for downstream machine learning classifier development and evaluation. 

### 2.2. Proteograph^TM^-Enabled Proteomic Profiling of Serum Specimens

**Figure 2:**
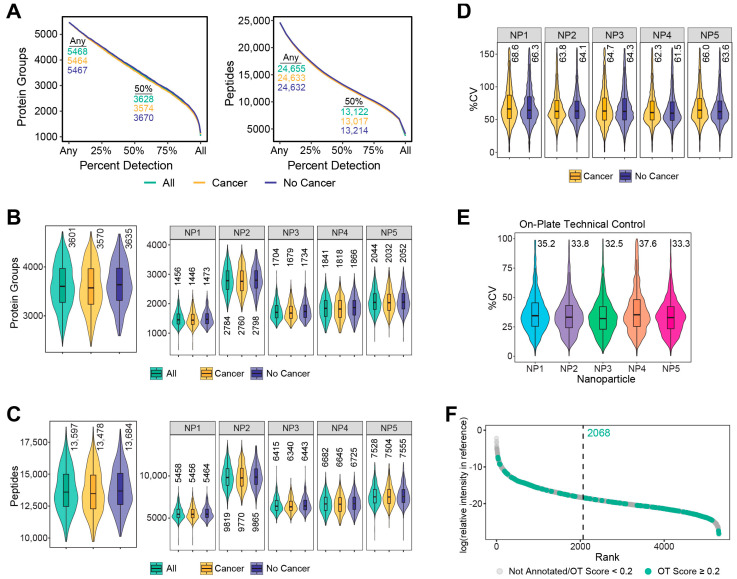
Proteomic characterization of subjects from serum prepared through Proteograph^TM^. (**A**) The number of protein groups and peptides identified at a given detection percentage across all samples, including subjects with cancer and without cancer. Proteins and peptides identified for the entire study and at least 50% of subjects are indicated on the plot. Distribution of the number of protein groups (**B**) and peptides (**C**) identified for all subjects, including those with cancer and without cancer, from any number of NPs (**left**) or per NP (**right**). Inner boxplots show the 25, 50, and 75th percentile of data, with whiskers representing ±1.5 × interquartile range (IQR). Median protein groups or peptides are indicated above each plot. Violins encompass all observations. (**D**) Distribution of percent coefficient of variation (%CV) calculated from MaxLFQ values for each protein group per NP across cancer and no cancer subjects. Missing values are not included in the calculation. Boxplots display the same information as above except outliers, defined as equal to or beyond the IQR, which are not shown. Outer violins only capture data points up to the limits shown on the axis. (**E**) %CV for proteins enriched for the PC3 technical control across all batches of samples per NP. Parameters for the plot are the same as previously described. (**F**) Protein groups observed in any NP across all subjects are matched to plasma proteins characterized in Keshishian et al. [40] and their respective relative intensity and sorted rank based on published concentration. The median rank for these matched identifications is displayed by the vertical dashed line. Identified proteins matching those in the Open Targets database for prostate cancer with a globalScore of ≥0.2 are colored in green, while those with a globalScore < 0.2 or not matching any proteins in the list are in grey.

Serum from the study populations was processed using Seer Proteograph^TM^, and resulting peptides from the 5-nanoparticle enrichment were analyzed through label-free dia-PASEF MS (Figure 1). No samples were removed upon analysis for outlier protein group identifications, which were defined for this study as <25% of the average number of proteins identified across all samples. At the time of writing, this study represents one of the largest MS proteomic discovery studies in serum. Across all samples, 5468 protein groups were identified in at least one subject from 24,655 total peptides, and 3628 of those protein groups were detected in at least 50% of the subjects, corresponding to 13,122 peptides (Figure 2A). The median number of protein groups detected per subject across any of the five nanoparticles was 3601, with the nanoparticle-specific enrichments ranging from 1456 to 2784 median protein groups identified (Figure 2B). Correspondingly, the median peptides per sample was 13,597 (Figure 2C). Individual nanoparticles varied from 5458 to as high as 9819 median peptides identified across all specimens. Subjects showed similar numbers of protein or peptides detected regardless of cancer status (Figure 2A–C). The percent coefficient of variation (%CV) was determined for the unnormalized MaxLFQ intensities [39] for each protein group per nanoparticle across each of the subjects separated by cancer status. The distribution of %CV, composed of both biological and technical sources of variability, is displayed as violin plot–boxplots in Figure 2D. The median protein intensity %CV was <69% for each of the nanoparticles. Peptide level %CV was equivalent to that observed for protein intensities (Figure S3). The technical variability of the study, which includes sources due to sample preparation and MS acquisition, was estimated using an on-deck process control present on each plate of 15 samples across the entirety of the study. The results from this process control showed that the median estimated technical variability was ~35% (Figure 2E). Protein groups identified in these subjects were mapped back to a deep plasma proteome dataset from Keshishian et al. [40] and showed broad-based coverage across the dynamic range of the blood proteome, with many of these detected proteins annotated in the Open Targets database for prostate cancer (Figure 2F) [41].

### 2.3. Differential Protein Expression across Cancer Subgroups and No Cancer Controls

**Figure 3:**
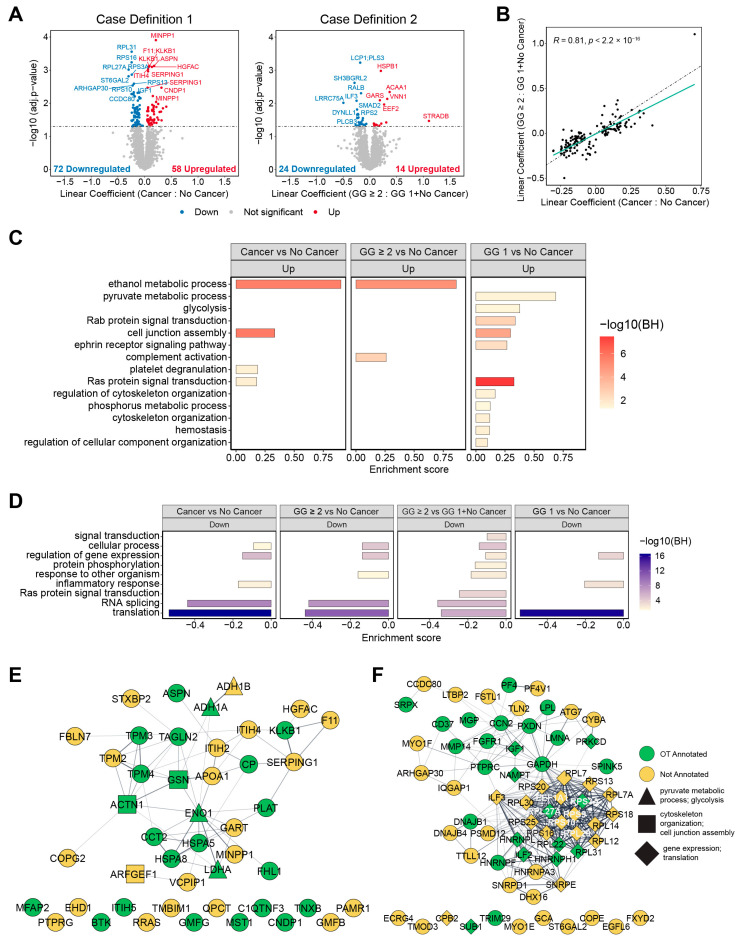
Differential protein expression for case definitions 1 and 2 and resulting functional enrichment analysis. (**A**) Volcano plots showing differential protein abundance from a linear mixed model for case definition 1 (**left**) and case definition 2 (**right**). Linear coefficients represent the log2 fold change in protein intensity between the respective indicated groups. (**B**) Scatterplot comparing the log2 fold change linear coefficients of protein groups with statistically significant (adjusted *p*-value < 0.05) differential abundance in case definition 1 vs. case definition 2. The dashed line in black, y = x, represents R = 1 correlation between the log2 fold changes in protein abundance and the solid line in teal is the resulting linear regression fit to the displayed data. The Pearson correlation coefficient, R, between these fold changes is displayed in the plot. (**C**) Upregulated and (**D**) downregulated processes according to 1D annotation enrichment analysis for different combinations of cancer and no cancer comparisons. (**E**) Interactome of the upregulated proteins and the downregulated proteins (**F**) for case definition 1. Edges represent interactions with a minimum STRING overall confidence score of 0.4.

Following normalization to adjust for technical variability, a linear mixed model was used to determine differential protein expression between subjects with cancer vs. no cancer (case definition 1) and GG ≥ 2 vs. GG 1 plus no cancer (case definition 2). The model also adjusted for batch effects and for the extracellular content of a subject’s serum (Section 4.9). For case definition 1, there were 130 protein groups with statistically significant differential expression (FDR < 0.05), including 58 upregulated and 72 downregulated (Figure 3A). Case definition 2 showed fewer differentially abundant protein groups, 38, with 14 and 24 of those up- and downregulated, respectively. Only 10 common protein groups were differentially abundant in both case definitions, but agreement in fold change for all proteins that were statistically significant across either comparison showed high correlation, R = 0.81 (Figure 3B). Functional enrichment analysis via 1D annotation enrichment was performed on the genes mapped to the protein groups in the dataset (Table S3) [42]. Selected biological processes meeting a significance threshold of FDR < 0.05 are shown in Figure 3C for upregulated enriched pathways across various cancer subtype comparisons and Figure 3D for those downregulated. Interaction networks from STRING [43] with highlighted proteins annotated for prostate cancer in the Open Targets database are displayed for proteins with upregulated (Figure 3E) and downregulated (Figure 3F) abundances. Enriched upregulated processes are observed for cytoskeletal organization and its regulation, which has known involvement in prostate cancer progression and proliferation specifically through its role in the epithelial-to-mesenchymal transition [44] and several metabolic pathways, such as glycolysis and pyruvate metabolism. Processes associated with gene regulation and translation were downregulated in multiple cancer subtypes relative to the no cancer subjects. Proteins annotated for these processes are indicated by different node shapes in the interactomes.

### 2.4. Protein Multimarker (MM) Classifier for Prostate Cancer Diagnosis 

A custom machine learning pipeline was developed to build a multimarker signature from the protein intensities for each nanoparticle in the training set. After data preprocessing, protein–nanoparticle features were filtered based on ranked *p*-values from a Wilcoxon rank sum test and pairwise correlation and then combined into a random forest classifier (Section 4.10.2). We focused our classifier building on the subset of subjects with PSA 4–10 ng/mL, as these are the intended population for PSA reflex tests. Data from subjects meeting this criterion in the training set were used to build a classifier separately for cancer vs. no cancer (case definition 1) and GG ≥ 2 vs. both GG 1 and no cancer (case definition 2). Optimization of the parameters for data preprocessing and feature selection was performed exclusively in this training set (Figures S8 and S9). This model was evaluated against those generated from AutoML, an automated machine learning pipeline that uses a Bayesian framework for model selection and hyperparameter tuning. AutoML was used separately on the entirety of the training data and to replace random forest as the classifier within our custom pipeline. This was performed to assess our custom pipeline for bias and overfitting (Figures S10 and S11, and Table S4). The protein multimarker signature for our optimized classifier achieved an average AUC of 0.64 (95% CI: 0.58–0.70) for case definition 1 using cross-validation compared to 0.60 (95% CI: 0.54–0.67) for the Prostate Cancer Prevention Trial (PCPT) risk calculator version 1.0 [35], which served as our clinical benchmark in the same set of patients. PCPT utilizes clinical factors, encompassing subject age, race, family history, DRE, and prior biopsy results in addition to PSA measurement to determine an assessment of risk for prostate cancer. For case definition 2, we observed an average AUC of 0.62 (95% CI: 0.56–0.68) with the protein feature classifier versus 0.60 (95% CI: 0.52–0.67) with the PCPT risk score. Full details of classifier performance are summarized in Table 2.

### 2.5. Validation of the Multimarker Signature

We next sought to validate the protein multimarker signature from our custom pipeline in the withheld validation set. The performance of the protein feature classifier for case definition 1 was 0.48 (95% CI: 0.39–0.58) for AUC when evaluated in the reserved validation set compared to the PCPT risk score of 0.70 (95% CI: 0.61–0.79), with a one-sided *p*-value of 1.0 (Table 2 and Table S5). Similarly, the protein feature classifier for case definition 2 was unable to surpass the performance of the clinical benchmark, reaching an AUC of 0.51 (95% CI: 0.40–0.62) versus 0.68 (95% CI: 0.58–0.78) for PCPT; the one-sided *p*-value was 0.99.

### 2.6. Bootstrapped Bias-Corrected Cross-Validation

**Figure 4:**
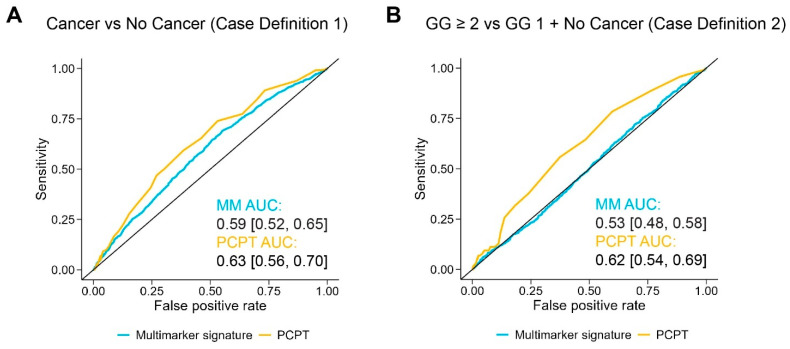
Classifier performance of the protein multimarker signature. ROC curves are shown for the protein-level data and the PCPT risk score in subjects with PSA 4–10 ng/mL for cancer vs. no cancer, defined here as case definition 1 (**A**) and GG ≥ 2 vs. both GG 1 and no cancer, case definition 2 (**B**). Average AUC for the protein signature and the PCPT risk score are displayed for each respective plot, with the 95% confidence interval indicated in brackets.

Given the protein multimarker signature in the held-out validation set did not surpass the clinical benchmark, we combined samples from the training and validation sets to utilize the entirety of the study population and maximize the amount of data available to train the protein classifier. Along with considering only individuals in the PSA 4–10 ng/mL range, we also developed models using all subjects. To reduce bias and overestimation of out-of-sample performance from the selection of model hyperparameters outside of a fully nested cross-validation scheme, we utilized bootstrapped bias-corrected cross-validation (BBC-CV) [45] to select optimal model configurations, estimate out-of-sample performance with bootstrapping, and provide a measure of the algorithm’s ability in the selection, fitting, and tuning of the model (Figure S13). Details of the BBC-CV employed are further described in Section 4.11.2. As displayed in Figure 4, the performance of the protein multimarker signature in subjects restricted to PSA 4–10 ng/mL for case definition 1 as estimated using the BBC-CV was 0.59 (95% CI: 0.52–0.65) for AUC. For the PCPT risk score, the AUC was 0.63 (95% CI: 0.56–0.70). In case definition 2, the protein multimarker signature achieved an AUC of 0.53 (95% CI: 0.48–0.58) compared to 0.62 (95% CI: 0.54–0.69) for PCPT. Similarly, a classifier generated for all subjects approached but did not exceed the diagnostic performance of the PCPT benchmark (Table 2 and Figure S12).

### 2.7. Top Protein Features in the Classifier

Protein features from the BBC-CV were scored based on their frequency of inclusion in a cross-validation fold for a selected model and its variable importance as defined by R package caret for random forest (further described in Section 4.12). The top 100 features ranked by the summary variable importance score for each classifier (case definition 1 or 2, all subjects or PSA 4–10 ng/mL) were compared to their respective set of statistically significant (adjusted *p*-value < 0.05) differentially abundant proteins from the linear mixed model (Figure S14A). Overlap in protein groups was highest for case definition 1 using all subjects with 22 protein groups shared across the top 100 features from the classifier and those from the 130 in the linear model. Several of the highest-scoring features from the classifiers include those from protein groups highlighted in the differential expression analysis, including blood markers CPB2 and IGF1, as well as proteins involved in metabolism, such as ENO1 and LDHA (Tables S6 and S7).

## 3. Discussion

In our patient serum discovery study, we identified novel proteomic blood biomarker candidates supporting downstream development of clinical tests aimed at improving prostate cancer detection prior to prostate biopsy decisions. Endpoint clinical assays, termed PSA reflex tests, provides additional assurance of prostate cancer’s presence beyond that of PSA testing alone, thereby reducing the number of unnecessary prostate biopsies. Leveraging two advanced, high-sensitivity platforms, Seer Proteograph^TM^ and trapped ion mobility mass spectrometry (MS), we conducted deep proteomic profiling in a large patient serum cohort comprising men referred for biopsy due to elevated PSA levels. The cohort was selected to maximize the clinical relevance of our findings and differentiates our study from those that use healthy men as controls. We achieved unprecedented serum proteomic depth in a large-scale prostate cancer discovery study. Across the 922 specimens profiled in this study, we observed greater than 3600 protein groups, even with a stringent 50% across-sample minimum detection limit (Figure 2A). From the 5468 total proteins identified in this study, there was broad coverage across the >10-order blood proteomic dynamic expression range, with a significant proportion demonstrating associations with prostate cancer based on curated ontologies (Figure 2F). 

We observed multiple differentially abundant proteins (adjusted *p*-value < 0.05) previously identified as prostate cancer blood markers when comparing subjects with any prostate cancer grades versus those with no cancer (Tables S1 and S2). These include the secreted ligand insulin-like growth factor (IGF-1) [46], proteins known to be elevated in prostate cancer patient serum, such as inter-alpha trypsin inhibitor heavy chains 2 (ITIH2) [47,48] and 4 (ITIH4) [48], apolipoprotein A1 APOA1 [48,49], carnosine dipeptidase (CNDP1), whose folding state has been demonstrated to be altered in prostate cancer patient plasma [50], and DEFA1B/DEFA3, a defensin protein(s) whose cognate transcript levels in blood predict prostate cancer treatment response [51]. Proteins with higher cancer abundance in this dataset that have previously been implicated in prostate cancer progression but not yet as blood markers include the coatamer protein COPG2 [52], oncogenic signaling protein BTK [53], and plasminogen activator PLAT, a known downstream gene target of ERG in tissues positive for the TMPRSS2–ERG fusion [54]. PSA (KLK3) itself was not detected in our proteomic dataset, perhaps representing a protein that is not enriched on the nanoparticle panel employed here due to specific physicochemical reasons.

To bioinformatically characterize the broad set of proteins differentially abundant in our study, we employed pathway enrichment using 1D annotation enrichment and network analysis. These approaches highlighted functions related to cytoskeletal organization, including activities specifically associated with prostate cancer progression and the mechanisms by which proteins may enter circulation (Figure 3C,E and Figure S6). Noteworthy proteins upregulated in our dataset (case definition 1) and in the prostate cancer literature included cofilin 1 (CFL1) [55] and Brefeldin-A-inhibited guanine nucleotide-exchange protein 1 (ARFGEF1) [56,57]. Similarly, alpha-actinin-1 (ACTN1) demonstrates significantly higher expression in malignant versus non-malignant African-American-derived prostate epithelial cell lines [58]. A final notable member, actin-binding protein gelsolin (GSN) [59], has been implicated in neuroendocrine transdifferentiation of prostate cancer [60].

In patients with lower-grade GG 1 cancer compared to those without cancer, we also detected enriched pathways indicative of blood coagulation that suggest a potential relationship to generalized coagulopathy and specifically disseminated intravascular coagulation, a condition known to be associated with advanced prostate cancer [61]. Another significant finding was the upregulation of glycolytic and pyruvate metabolic activities (Figure 3C,E and Figure S6), which have been indicated as primary energy sources for prostate cancer cells [62]. One exemplar protein of this metabolic pathway enrichment, enolase 1 (ENO1), converts 2-phosphoglycerate to phosphoenolpyruvate and serves as a cell surface receptor for plasminogen [63], where it is potentially involved in prostate cancer cell migration [64]. Additionally, lactate dehydrogenase A (LDHA) chain has shown overexpression in prostate tumors [65].

Finally, we detected significant enrichment for ribosomal proteins (Figure 3D,F and Figure S7) and related processes, such as translation and gene expression, but with higher differential abundance in blood from patients referred for biopsy but for whom no cancer was detected. While unexpected given the directionality [66], these candidates warrant further exploration given that several (RPL7A, RPS13, RPS18, RPS9) were among the top-scored features from the BBC-CV for classifier performance (Tables S6 and S7). Relatedly, spatial, single-cell RNAseq profiling from a study by Heidegger et al. also showed unexpected downregulation in processes normally ascribed to cancer, such as proliferation and signaling, when comparing adjacent tumor and normal epithelia, suggesting that some biological signal in cancer, such as those we observe here, may be related to secondary effects of the tumor microenvironment and not the tumor epithelia [67].

Protein features and their associated MS intensities were also used to train a multimarker machine learning classifier to assess diagnostic power versus a current standard-of-care benchmark, the PCPT risk calculator. In subjects with PSA levels between 4 and 10 ng/mL, where unmet need is most profound for new diagnostics, performance of the protein classifier in the training set exceeded that of PCPT for case definition 1 and was slightly below for definition 2. Despite the promise of this initial performance, formal validation in the withheld set did not recapitulate these results (Table 2). In subsequent analysis, training and validation sets were combined to increase statistical power. Here, the multimarker classifier achieved diagnostic performance slightly below PCPT for case definition 1 but provided no discrimination in classifying the relatively small subset of GG ≥ 2 subjects for case definition 2 (Table 2, Figure 4, and Figure S12). Removing the PSA restriction did not significantly change outputs. No additive performance was gained by combining PCPT risk scores with the proteomic signatures, nor was performance improved by analyzing datasets at the peptide level. Comparable performance levels were achieved in the training set using an independent machine learning pipeline, AutoML, to validate outputs of this study’s custom pipeline (Figure S11 and Table S4). We consider several factors that may have negatively impacted classifier performance. These include low overall fold changes, known to negatively impact machine learning classifier performance [68], and our use of a ‘no cancer’ comparator where all men demonstrated prostate abnormalities, principally elevated PSA, but no cancer detected. The latter may have been particularly impactful as this group may have more heterogeneous protein expression and overlapping, confounding signal between prostate abnormality and cancer compared to healthy, age-matched men without elevated PSA, a less relevant comparator class for our clinical endpoint. Notably, the abundance differences in more discrete comparisons (GG 1 versus no cancer and GG ≥ 2 versus no cancer, Figure S5B) appear heterogenous among subsets of proteins that display negatively correlative abundance changes, potentially dampening the overall signal in the broader comparison. Finally, while consistent EDRN collection protocols were uniformly employed, we detected multiple differences in proteomic outputs between the Finasteride and SABOR subcohorts (Figure S4). These were perhaps related to sample storage times at −80 °C, which were substantially longer for SABOR specimens but were also potentially affected by differences in recruitment criteria. Classifier analyses stratified by cohort, however, did not show substantively different performance across cohorts.

In summary, we identified a rich set of differentially abundant proteins but achieved diagnostic classifier performance for proteomic signatures not exceeding the clinical standard-of-care benchmark, with the latter demonstrating the considerable challenge of clinical proteomic biomarker discovery and operating at this stage of the translational diagnostic pipeline, where early cancer blood signals are typically subtle. We note the application of novel proteomic technologies and an approach to study design centered around a highly scaled, specially curated set of study specimens matched to the intended use case population. These choices of cohort inclusion criteria and study parameters were intentionally aimed towards identifying clinically relevant PSA reflex blood markers but potentially precluded the inclusion of more easily detected, but likely less useful, protein biomarkers. Achieving diagnostic power sufficient for meaningful clinical impact will assuredly require results from multiple complementary proteomic discovery studies similar to this one, including those employing different designs and cohorts [69,70,71], consideration of protein posttranslational modifications [49], combinations of multiomic features, as in the Stockholm-3 Test [72], and/or nomograms combining blood testing and MRI imaging [73]. Despite the challenges of cancer early detection, new proteomic technology, such as Proteograph, will be essential in enabling highly scaled biomarker discovery studies with deeper and more comprehensive profiling of the blood proteome that, along with parallel technologic developments in protein detection, should unlock the potential of employing proteomic markers for more effective early cancer diagnostics.

## 4. Materials and Methods

### 4.1. Study Design

In the PRoBE study design, specimens are collected prospectively from a population that resembles the target population for clinical application. Cases and controls are processed and analyzed in a fashion that is blinded to their status, and markers are evaluated based on metrics that are clinically relevant for the intended application. To minimize technical variance, block randomization for specimen preparation and data acquisition run orders were employed. To avoid operator bias, all individuals directly involved in preparing samples and/or acquiring MS datasets were blinded to clinical metadata until all specimen preparations and data acquisitions were completed. Finally, to minimize bias in estimating the performance of the multimarker signature, we reserved 30% of the data for an independent validation set. As a contingency plan, if the validation was underpowered, we evaluated the signature performance using cross-validation.

### 4.2. Study Population

We used serum samples from two cohorts that collected blood/serum samples from men who subsequently underwent prostate biopsies. Both cohorts used sample handling and storage protocols from the Early Detection Research Network (EDRN). The first cohort, the Finasteride Challenge Study (FCS) [37], consisted of participants in a trial investigating the effect of a 3-month dosage of Finasteride on biomarkers predicting the outcome of a prostate biopsy. For the purposes of this study, baseline serum samples were used to exclude Finasteride contamination. Between 2011 and 2016, FCS enrolled men from this cohort 55 years or older without prior history of prostate cancer who had an estimated risk of prostate cancer between 20 and 60% based upon the Prostate Cancer Prevention Trial (PCPT) risk calculator version 1.0 [35]. At 90 ± 14 days, subjects underwent a 12-core prostate biopsy. Each core was fixed in formalin, processed for pathologic assessment, and individually read for the presence of prostate cancer. After biopsy, subjects were followed clinically using the local standard of care.

We also drew serum specimens from UT Health San Antonio’s SABOR (San Antonio Center of Biomarkers for Risk for Prostate Cancer) cohort [38]. SABOR is a multi-ethnic, multi-racial cohort of 4170 men from San Antonio and South Texas with no prior diagnosis of prostate cancer who agreed to undergo routine prostate cancer screening at the time of enrollment. Between 2000 and 2011, subjects were seen annually for PSA testing and DRE, with an average of approximately 6 visits per participant. Since 2011, subjects were followed with PSA only, and those with PSA less than 1.0 ng/mL were seen biennially. Biopsies were performed and evaluated according to community standards. Included in our sample were all men in the SABOR cohort who had a prostate biopsy during the course of their participation, excluding the small subgroup of men who requested a biopsy without clinical indication. For men in our study, we used the serum sample collected most proximally (within six months of) and prior to the first biopsy in the sample. If the serum corresponding to the first biopsy was not available, we used the serum corresponding to the second biopsy.

### 4.3. Cancer Outcomes

In both cohorts, cancer cases are defined based on having a diagnosis of prostate cancer within 18 months of the index biopsy evaluation. The Gleason group (GG) was obtained based on the most recent biopsy within 18 months of the blood draw. In the FCS, we had data on GG on prostatectomy if the patient had one within 18 months of the blood draw (n = 20). For our multiple marker signature and differential expression analyses, we considered two case definitions: (1) cancer versus no cancer and (2) clinically significant cancer (GG 2 or higher) versus low-grade (GG 1) or no cancer.

### 4.4. Preparation and Batch Randomization

Subject blood samples were drawn at UTHSA in outpatient clinics for the FCS cohort and in the community before transport to UTHSA on ice for the first five years of SABOR before transitioning for follow-up at UTHSA clinics by year 5 and onwards. The maximum time between draw and serum preparation was 4 h. All subject serum specimens were prepared at a common site (UTHSA) by trained operators using unified EDRN protocols [36]. All serum specimens selected for this study had a maximum of one freeze–thaw cycle during storage at UTHSA. Serum aliquots and associated clinical metadata were transferred from UTHSA to OHSU via the CEDAR (Cancer Early Detection Advanced Research Center) Specimen Biorepository with pre-assignments to the order and batching of the Proteograph^TM^ and mass spectrometry processing. Those handling the specimen or sample derivatives were blinded to the case/control (cancer/no cancer) status at all steps in the process.

Samples were processed in batches of 16 (15 experimental samples, 1 processing control, described in more detail below). To minimize systematic bias from batch effects, we used a stratified randomization procedure to assign case and control samples to each batch, ensuring that approximately equal numbers of cases/controls appeared in each block. Cases and controls within a block were randomly assigned. Prior to finalization of the randomization list, we examined the distribution of age at biopsy, PSA, cohort, and race/ethnicity across the batches and rerandomized if there were obvious imbalances in these variables across the batches.

We also used stratified randomization to assign 70% of specimens to training and 30% to internal validation sets. The randomization was stratified by case/control status, race/ethnicity, age, and PSA to ensure proportionally equal representation of these variables in training and validation sets. The training set was intended to be used for development of a multimarker panel. The validation was intended to be used as an independent test set to assess the performance of the multimarker panel.

### 4.5. Automated Blood Serum Sample Processing with Proteograph^TM^ Workflow

Serum specimens (N = 922 from both cohorts) and control samples were prepared on two SP100 automation instruments with a Proteograph^TM^ Assay Kit included in the Proteograph Product Suit (Seer, Inc., Redwood City, CA, USA) using five distinctly functionalized nanoparticles (NPs) as previously described [22,29], with specific details as follows. Each Proteograph plate accommodated 16 samples and 3 fixed, on-deck control samples (Process, Digest, and MPE Controls). Specific to this study, 1 of the 16 sample slots on each plate was reserved for analysis of PC3, a pooled plasma sample that served as an additional end-to-end workflow technical control. Upon thawing of serum specimens in advance of Proteograph preparation, qualitative observations regarding specimen color and turbidity were recorded. Immediately after SP100 on-deck dispensing of serum specimens for nanoparticle enrichment, residual serum from the original 250 μL input was manually collected and stored at −80 °C. Following completion of SP100 Automation Instrument on-deck peptide cleanup and elution, purified peptides were quantitated using the Pierce Quantitative Fluorometric Peptide Assay (Thermo Fisher Scientific, Waltham, MA, USA) using an on-deck SP100 routine and a standard plate reader instrument (Spark20M, Tecan, Männedorf, Switzerland). Samples with outlier peptide recovery, defined as greater than 2-fold or less than 0.5-fold the ratio of sample peptide mass to median peptide mass for all samples on the plate for the corresponding nanoparticle, were flagged for possible preparative issues and subsequent re-processing with a second aliquot of serum. To further assess preparation quality control, 65 μL of the 3 on-deck control samples was dried to completion in LowBind Eppendorf tubes and analyzed through mass spectrometry (described below). Elutions from all 80 sample NP enrichments and the remainder of on-deck control samples for each plate were transferred to a 96-well shallow plate, dried to completion overnight in a vacuum concentrator, and then frozen at −80 °C. This sample preparation routine was employed for individual analysis of all 922 patient specimens as well as for preparation of pooled patient specimen serum samples, either from residual collection or from supplemental patient serum aliquots when available, to support generation of a study-specific spectral library (described in more detail below).

### 4.6. Data-Independent Acquisition (DIA) Liquid Chromatography-Coupled Mass Spectrometry (LC-MS)

In real-time, during sample preparations using Proteograph^TM^, the three standard on-deck controls were subjected to label-free dia-PASEF (data-independent acquisition parallel accumulation serial fragmentation)-mode liquid chromatography–mass spectrometry (LC-MS) in positive mode using a timsTOF Pro MS system (Bruker, Billerica, MA, USA), CaptiveSpray source (Bruker), and an inline-coupled nanoElute liquid chromatography instrument (Bruker). This served to conduct a proteomics assessment of the automated Proteograph workflow with minimal delay to avoid the further loss of subsequent samples due to problems with sample processing that were not obvious solely from peptide recovery. The timsTOF *m*/*z* and tims dimensions were linearly calibrated before each batch of samples or every 4–5 days for continual acquisitions using 3 selected ions from the Agilent ESI LC-MS tuning mix [*m*/*z*, 1/K0: (622.0290, 0.9915), (922.0098, 1.1986), (1221.9906, 1.3934). Dried control samples were resuspended manually in 2% ACN and 0.1% FA containing 5 pmol/μL of PepCal standard peptide mixture (Promega, Madison, WI, USA) for a final peptide concentration of 200 ng/μL and loaded into autosampler vials. For dia-PASEF, the MS scan range was 100–1700 *m*/*z*, the ion mobility window was 1/K0 0.6–1.6, the ramp and accumulation time was 100 ms, the source voltage was 1700 V, the dry gas was 3.0-L/min, and the dry temperature was 200 °C. The mobilogram shape and ion mobility window (1/K0 0.6–1.6) were tailored to Proteograph enrichments of serum. MS-coupled nanoflow reversed-phase chromatography at a flow rate of 0.5 μL/min was accomplished on the nanoElute instrument using mobile phase Solvents A (water, 0.1% formic acid) and B (acetonitrile, 0.1% formic acid), an inline PepMap C100 C18 cartridge trap (5 mm/0.3 mm/5 µm particle, Thermo Fisher Scientific), a C18 analytic column (15 cm × 150 µm × 1.5 µm particle size, PepSep, Marslev, Denmark) housed in a Column Toaster (Bruker) at 50 °C, and a 20 μm Classic Emitter (Bruker) with a 43 min total time acquisition (0 min, 5%B/28 min, 35%B/35 min, 40%B/36.67 min, 95%B/43 min, 95%). Baseline proteomic outputs for protein group and peptide identifications were initially set with 8 injections of the 3 Proteograph technical control types run on healthy serum controls. In concert with measurements of peptide recovery, peptide and protein ID rates of the 3 controls based on library-free database searches using DIA-NN [74] (details below in Section 4.8) were monitored on PAS and observed for drops below 2 × sd (standard deviation) from the mean (Figure S2). LC-MS performance of the instrument measuring these control samples was assessed every set of two plates with a K562 system suitability sample for numbers of protein groups and peptides. Deviations of either of these metrics below 2 × sd triggered an assessment of the instrument. Additionally, reconstructed peaks for PepCal spike-in peptides were also monitored qualitatively with Panorama AutoQC 21.1.0.158 [75]. 

For interrogation of Proteograph enrichments from cohort specimens and the PC3 control, DIA LC-MS was performed on individual nanoparticle enrichments from each specimen with separate injections. These acquisitions were performed using label-free dia-PASEF-mode LC-MS using two additional timsTOF Pro MS platforms, each coupled to an UltiMate 3000 nanoLC instrument (Thermo Fisher Scientific). Dried peptides from Proteograph enrichments were reconstituted in 2% ACN, 0.1% FA containing 5 pmol/μL of PepCal standard (Promega) to a final peptide concentration of 200 ng/μL using an on-deck Proteograph SP100 routine. Peptide (200 ng) from each NP enrichment was analyzed per injection. Nanoparticles 1–3 were analyzed on one timsTOF and 4–5 on the other to minimize technical MS acquisition bias across specimens. A standard dia-PASEF method was used with settings identical to those indicated above, except the ion mobility range was set to 1/K0 0.6–1.4 and a smaller mobilogram was used for faster cycle time. MS-coupled reversed-phase chromatography was accomplished using mobile phase A (water, 0.1% formic acid) and B (acetonitrile, 0.1% formic acid), an inline cartridge C18 trap (Thermo Fisher Scientific), a micropillar array C18 column (50 cm uPAC C18, PharmaFluidics, Gent, Belgium), and a 20 µm Classic Emitter (Bruker) with a 33 min total time method. Quality control of MS acquisitions (>4500 injections for the entire cohort analyzed on a per-nanoparticle basis) included assessment of peptide and protein identification rates from library-free DIA-NN database searches, determination of retention times and chromatographic profiles for reconstructed PepCal peaks using Skyline 21.1 and Panorama AutoQC 21.1.0.158 [75] and Compass DataAnalysis (Bruker), as well as monitoring of total intensity chromatograms and LC backpressures. A small subset of samples with issues flagged by the above quality control checks was prepared again from remaining serum aliquots and re-analyzed. For one sample, subject 476, acquisitions for NP1 and NP4 were unable to be completed and were not included in subsequent data analysis.

### 4.7. Spectral Library Generation

A subset of specimens from the two-cohort training set was selected as input material for spectral library assembly. First, training set serum samples exhibiting distinguishing color and/or turbidity (n = 18, the “qualitatively different” or “QD” samples) as detected through qualitative observation upon thawing were selected for spectral library generation. A second subset of the remaining training set specimens (“qualitatively normal” or “QN” samples) comprising the majority of the specimens were chosen as follows. During progression of DIA LC-MS acquisition for all cohort specimens, emerging raw data were monitored in real-time using library-free DIA-NN through the Seer cloud-based Proteograph^TM^ Analysis Suite (PAS, Seer, Inc.) to determine aggregate unique protein and peptide identifications and the contribution provided by the incremental specimen dataset addition. Saturation of new, unique peptide identifications (18,000–19,000 unique peptides) on resulting accumulation plots (Figure S1A) indicated that the subset of protein and peptide identifications observed through 450 samples would be approximately representative of the total proteomic diversity in the remainder of the cohort. Several approaches were tested for sample selection in the generation of a spectral library, including random sample selection and using maximal distance between selected samples from principal component analysis (PCA). A custom “MaxID” algorithm was developed, which identifies the samples that will provide the maximum number of possible peptide identifications based on the set of interrogated samples (deposited in GitHub on 8 July 2024 at https://github.com/CEDAR-Proteomics/Orion, Figure S1B). The same method can also be used to model the total number of peptides identified from a given number of samples using the MaxID method, allowing for the determination of the desired sample size used as input for the spectral library (Figure S1C). A targeted subset of 75 QN specimens was prioritized for spectral library inputs using this algorithm to reasonably represent overall cohort proteomic diversity. 

Several approaches were tested for spectral library generation using these selected samples, and an approach using deep offline fractionation provided the deepest library. In the primary approach, pools of peptides for the QD and QN samples were generated with the five nanoparticle enrichments for each kept separate (10 pools total). To build these pools, both (a) peptides left over from initial Proteograph preparations and (b) supplementing peptides from second-round Proteograph preparations performed on additional serum aliquots of the same specimens provided by UTHSA were leveraged. For fractionation of these 10 resulting pools, offline, basic reversed-phase high-pressure liquid chromatography was performed by the University of Reno Las Vegas Proteomics Core Facility. Pooled samples submitted were dried down completely and reconstituted in 6 μL of Solvent A (10 mM of ammonium hydroxide in water, pH 10). Basic pH reversed-phase fractionation of 5 μL of this resuspension was performed on an UltiMate 3000 LC instrument (Thermo Fisher Scientific) fitted with an Acquity UPLC BEH C18 column (100 mm × 1.0 mm × 1.7 μm particle size, Waters, Milford, MA, USA) using a flow rate of 75 μL/min with an increasing gradient of Solvent B (10 mM of ammonium hydroxide in acetonitrile, pH 10; 2–55% over 47 min, total time 62 min). Fractions were collected every 50 s for 62 min and concatenated to 24 aggregate fractions for each peptide pool. All concatenated fractions were returned to OHSU, dried to completion, and resuspended in 7 μL of MS-grade water, 0.1% formic acid. 

Each reconstituted fraction was analyzed through LC-MS using data-dependent acquisition parallel accumulation serial fragmentation (DDA-PASEF) [31] on a timsTOF Pro1 MS system (Bruker) using a CaptiveSpray source (Bruker) and inline-coupled UltiMate 3000 liquid chromatography instrument equipped for nanoflow (Thermo Fisher Scientific). For DDA-PASEF, the MS scan range was 100–1700 *m*/*z*, the ion mobility window was 1/K0 0.6–1.4, the ramp and accumulation time was 166 ms, the number of PASEF ramps = 8, the source voltage was 1700V, the dry gas was 3.0-L/min, and the dry temperature was 200 °C. MS-coupled nanoflow reversed-phase chromatography at a flow rate of 0.5 μL/min was accomplished on the nanoELUTE instrument using mobile phase Solvents A (water, 0.1% formic acid) and B (acetonitrile, 0.1% formic acid), an inline PepMap C100 C18 cartridge trap (5 mm/0.3 mm/5 μm particle, Thermo Fisher Scientific), a C18 analytic column (25 cm × 150 µm × 1.5 μm particle size, PepSep) housed in a column heater (MonoSLEEVE, Analytical Sales and Services, Flanders, NJ, USA) at 50 °C, and 20 µm Classic Emitter (Bruker) with a 120 min total time acquisition (0 min, 4%B/92 min, 30%B/102 min, 35%B/104 min, 95%B).

Serum from the same sample sets as used in the primary spectral library strategy detailed above were also pooled together in an alternative approach and processed with Proteograph. From these NP enrichments of pooled serum, peptides were analyzed through DDA-PASEF, as described above but using a 240 min total acquisition time. Library depth was compared to the first strategy, with fractionation providing the highest number of proteins and peptides. Ultimately, these were combined into one spectral library from both approaches for use in this study.

### 4.8. Raw MS Data Processing

Raw DIA MS (.d) data for each of the subjects and controls were searched in PAS with DIA-NN 1.8 against the canonical Uniprot human protein sequence fasta database with isoforms included (version from 2 March 2022). For DIA-NN library-free, default settings were used, except MBR was not enabled. The MS1 and MS2 Mass Accuracy were each set to 10 ppm. Precursor FDR (Q Value), Global Precursor FDR (Global Q Value), and Global Protein Group FDR (Global PG Q Value) were all 0.01. N-term M excision and C carbamidomethylation were set to True, while M oxidation and Acetyl (N-term) were False. For searches with the study-specific spectral library, the same parameters were used as described above, and Reannotate set to True.

To generate the study-specific spectral library, the MSFragger-DIA-NN workflow for spectral library generation was used [76]. FragPipe (version 18.0), MSFragger (version 3.5) [77], Philosopher (version 4.4.0) [78], and EasyPQP (version 0.1.30) were used to search the raw DDA-PASEF data files using the same fasta database employed above and to create a spectral library from the results. Reverse decoys were appended to the database using Fragpipe.

### 4.9. Differential Expression Analysis and Functional Annotation

Data processing and analysis were performed in R Statistical Software (version 4.2). Protein group intensities per NP quantified through MaxLFQ were log2-transformed and fold-change-normalized to mitigate technical variance across sample NP MS runs. For normalization, we initially identified protein groups present in at least 80% of the samples. Next, we computed the fold change per protein group per NP by subtracting the median intensity across samples from the intensity. Subsequently, the shift of fold change per sample per NP was determined as the fold change minus the median fold change across samples. Finally, we normalized the log2-transformed intensities of all proteins by subtracting the fold change shifts.

Protein–NP pairs absent in 60% or more of the samples were excluded (40% minimum sample detection), resulting in 3578 quantified protein groups eligible for differential analysis. Missing protein intensities were imputed for the NP with the highest number of non-missing intensity measurements for a given protein. The imputation process assumes that data are not missing at random, and that missing values are likely to be low values (below the limit of detection of the mass spectrometer). To that end, missing values were sampled from a normal distribution with parameters Normal $\left(I_{0}–1.8\sigma ,0.3\sigma \right)$, where $I_{0}$ represents the mean and σ denotes the standard deviation of protein log2 intensities in the entire dataset [79].

A linear mixed model was employed to identify proteins exhibiting significant up- or downregulation in cancer compared to non-cancer (case definition 1) or in high-grade compared to low-grade cancer (case definition 2). Additionally, a model incorporating a three-level version of the PC status variable (non-cancer, low-grade cancer, and high-grade cancer, with non-cancer serving as the reference group) was fitted. For brevity, we present the model with single case status coding for case definition 1, denoted as PCStatus. The lme4 (version 1.1-35.1) package was utilized to fit the model separately for each protein, pooling information across NPs.

Each model adjusted fixed effects, including age at serum collection, year of serum collection, outlier status of samples based on appearance, and sample composition. Sample composition was quantified as a continuous covariate that accounts for the variability of blood plasma subfractions enrichment in the NP protein corona of a given Proteograph^TM^ sample. This variability stems from various factors, such as sample collection methods and age-related changes in blood composition. The variable was calculated as the median log2 fold change (relative to the median protein log2 intensity across the cohort) of proteins annotated as belonging to the “Extracellular” GO Cellular Compartment in each Proteograph sample.

For each protein, given plate *i*, NP *j*, and subject *k*, the linear mixed effects model of the normalized log-intensity values $Y_{ijk}$ was as follows (omitting additional fixed effects for brevity):

(1) $$Y_{ijk}=\beta_{0}+\beta_{1}PCStatus_{ijk}+u_{i}+v_{j}+w_{ik}+z_{k\left(i\right)}+\epsilon_{ijk}$$

where $u_{i}$, $v_{j}$, $w_{ik}$, and $z_{k\left(i\right)}$ represent random effects for the plate, NP, plate × NP, and subject nested within the plate, respectively. For proteins with only a single NP having non-missing intensity values, the model excluded random effects $v_{j}$, $w_{ik}$, and $z_{k\left(i\right)}.$ This structure effectively captures the technical variance attributable to plate effects and accounts for the correlation between NP–protein pairs within individuals. The coefficient corresponding to the PCStatus variable represents the mean increase or decrease in fold-change-normalized log2 intensity by case status, adjusting for other covariates.

*p*-values associated with the estimated PCStatus coefficient, derived from the linear models for each protein group, were adjusted for multiple testing using the Benjamini–Hochberg method. Proteins with a corrected *p*-value below 0.05 were subjected to functional enrichment analysis using 1D annotation enrichment analysis [42]. These differentially abundant networks were further assembled into interaction networks with STRING 12.0 (accessed on 26 May 2024) [43] and further visualized in Cytoscape 3.10.2 [80].

### 4.10. Protein Multimarker Classifier

#### 4.10.1. Multimarker Signature Development

We developed two multimarker signatures, one to predict cancer presence vs. no cancer and one to predict GG ≥ 2 compared to no cancer/GG 1 cancer. We limited signature development to individuals with PSA levels 4–10 ng/mL, as they represent the target population for a prostate reflex test in clinical settings. The signatures incorporate individual features based on protein–NP pairs.

In developing the multimarker signature, various algorithmic approaches were considered. The first approach involved a custom pipeline that preprocesses the data, filters markers, and applies a classification algorithm to the chosen marker subset. Filtration aims to eliminate markers with poor coverage, high correlation with other markers, and no marginal association with case status. Within the custom pipeline, we considered two types of classification algorithms, random forest and AutoML, an ensemble learning algorithm utilizing Bayesian optimization to simultaneously search for optimal algorithms and hyperparameters [81,82]. In addition to the custom pipeline, we implemented standard AutoML on log2 MaxLFQ intensities to validate our custom pipeline against an approach that searches through a space of general preprocessing steps and classification algorithms. The random forest model was fit with the R package caret (version 6.0-94), and AutoML was fit with Python (version 3.9.16) using the package auto-sklearn (version 0.15.0) and scikit-learn (version 0.24.2). 

#### 4.10.2. Custom Pipeline

**Scheme 1:**
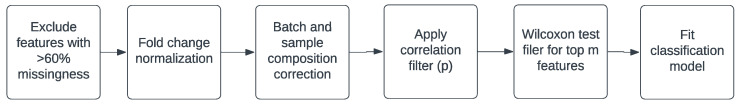
Overview of the steps of the machine learning pipeline employed.

The steps in the custom pipeline are illustrated in Scheme 1. Initially, features with more than 60% missingness were excluded. Subsequently, the data were fold-change-normalized using the previously described method. Next, the fold-change-normalized log2 intensities were adjusted for batch effects and sample composition by fitting the linear mixed model

(2) $$Y_{ik}=\beta_{0}+u_{i}+SampleComposition_{ik}+\epsilon_{ik},$$

where $Y_{ik}$ represents the fold-change-normalized protein–NP intensity for plate i and subject k, $u_{i}$ represents random effects for the plate, and $SampleComposition_{ik}$ is the sample composition covariate computed as previously described. The residuals obtained from this model replaced the log2 max-LFQ intensity as features in the subsequent stages of the pipeline. Finally, missing values in transformed intensities were imputed with zero imputation, under the assumption that data are not missing at random and that missing values are not present in the sample.

Following pre-processing, features undergo ranking based on Wilcoxon tests of differential expression in cases versus controls. Subsequently, pairwise correlations are computed among the top 1000 features. For a given feature, denoted as F, other features are identified whose correlation with F surpasses a predefined cutoff value, denoted as *p*. If any of these correlated features exhibit higher coverage than feature F, it is excluded from the set. The algorithm iterates through all features, retaining those that pass the filtering process. Post correlation filtering, the remaining top k features, identified through the Wilcoxon tests, are preserved. These remaining features are then utilized in the classifier.

#### 4.10.3. Model Selection and Tuning

We employed three performance metrics for model selection: (1) area under the receiver–operator curve (shown here as AUC), (2) average positive predictive value (PPV), and (3) average negative predictive value (NPV). PPV is equivalent to the area under the precision–recall (PPV–sensitivity) curve, reflecting the mean positive predictive value across various classification score cutoffs. NPV corresponds to the area under the NPV–specificity curve, representing the mean negative predictive value across a range of classification score cutoffs. The selection criteria were based on maximizing the sum of standardized cross-validation AUC, PPV, and NPV.

For the filtration component of the pipeline, the number of features included in the model, m, and the correlation cutoff, *p*, were evaluated using a nested cross-validation approach with 10 outer folds, using random forest as the classification method. For training portions of the outer folds, the pre-filtering pipeline was executed for specified values of m = 20, 40, 50, 80, 100, 125, 160, and 200 and *p* = 0.5, 0.6, 0.7, and 0.8, and internal 10-fold CV was used to tune the classification model. Predictions were then generated for the testing portions of the outer folds, and AUC, PPV, and NPV were calculated in testing portions. We repeated the CV 3 times for 3 different random seeds to minimize Monte Carlo error. We then selected the classification algorithm using a similar 10-fold nested CV, comparing filtering + random forest, filtering + AutoML, and AutoML alone. The filtering steps utilized previously optimized values of m and *p*.

After selection of m, *p*, and classification model type, hyperparameters specific to the classification model were then identified by standard CV on the full training data. Upon selection of all hyperparameters, the final model was obtained for the full test data. This final model was then used to generate predictions of case status in the withheld validation data.

### 4.11. Validation Analysis

#### 4.11.1. Assessment of the Classifier in the Held-Out Validation Set

In the withheld validation set, we computed the difference in AUC, NPV, and PPV for the PCPT risk score and protein model and hypothesis tests for statistical significance of these differences. The R packages pROC (version 1.18.5) and usefun (version 0.5.0) were used for one-sided hypothesis tests of differences in the AUC and NPV/PPV, respectively.

#### 4.11.2. Contingency Plan for Model Tuning and Estimation of Out-of-Sample Performance Using Bootstrapped Bias-Corrected Cross-Validation

Prior to conducting the validation, we devised a contingency plan to utilize the full data to estimate out-of-sample performance in case the formal validation analysis was underpowered, and there were no statistically significant differences in AUC, NPV, or PPV between the PCPT risk score and the multimarker signature.

In order to address optimism in cross-validation estimates of out-of-sample performance arising from selection of model hyperparameters outside of a fully nested cross-validation scheme, we adopted a bootstrapped bias-corrected cross-validation approach (BBC-CV) [45]. BBC-CV effectively utilizes distinct information to select optimal configurations and perform model evaluation, employing bootstrapping to obtain a distribution of estimates for out-of-sample performance. Ultimately, BBC-CV provides an evaluation of the overall algorithm’s performance in fitting, tuning, and selecting a model, rather than focusing on a specific model.

Operationally, in BBC-CV, each model configuration (i.e., a pipeline run under specific values of hyperparameters m and *p*) is trained and predicted on the same set of 10 cross-validation (CV) folds. Each CV was repeated across three separate random seeds to minimize Monte Carlo error. Predictions are aggregated across folds for each configuration and then repeatedly resampled with replacement. During each bootstrap iteration, performance metrics are assessed for the sampled rows, and the top-performing configuration is selected. In that iteration, the validation metrics are computed for the unsampled data based on the chosen configuration. Model performance is summarized based on the distribution of validation metrics on the unsampled partitions across the bootstrap replications. We used the strategy for model selection as previously described based on finding the configuration that maximizes the sum of standardized AUC, PPV, and NPV.

To summarize the out-of-sample performance of the signature, we presented BBC-CV estimates of AUC, PPV, and NPV. Additionally, the same statistics for the PCPT risk score, utilizing the same patients in the validation partition for each bootstrap iteration, were determined along with differences in AUC, PPV, and NPV between the protein signature and the PCPT risk score. Confidence intervals for these differences using the 2.5th and 97.5th percentiles of the distribution of these metrics across bootstrapped iterations were also calculated.

### 4.12. Summarizing Importance of Features in the Multimarker Signature

To summarize the feature importance of protein–NP features in the ML learning model, we reported on the features included in the model and the variable importance of the features. Caret determines variables’ importance in random forests by recording prediction accuracy on out-of-bag data for each tree, repeating the process after permuting each predictor variable, and, finally, averaging the standardized difference between prediction accuracy and permuted prediction accuracy over all trees. In the case that the true validation showed a significant improvement between the multimarker signature and the PCPT risk score, we planned to report on these statistics from the final model selected from the training set. In the case that the true validation failed to show a significant improvement, we planned to report on the mean of the average of these statistics across CV folds for a given model configuration weighted by the frequency of a given model configuration, which was selected in the BBC-CV.

## 5. Conclusions

Using two differentiating proteomic platforms, we identify a rich set of differentially abundant candidate proteins to support future clinical PSA reflex tests for improved prostate cancer diagnosis. As this discovery-mode effort sits at the very early stages of clinical test development, new biomarker candidates will require extensive validation in large and diverse patient cohorts to confirm their detection efficacy, prioritize marker subsets, and ensure the applicability of selected markers to broad patient populations. While the sophisticated technology platforms employed here are ideal for proteomic marker discovery, particularly in biofluids, such as blood, their complexity of operation and high cost likely preclude their use in the routine clinical setting. As such, in order to realistically support a feasible and widely adopted clinical test, a subset of validated and prioritized biomarkers from discovery-mode studies, such as this one, will almost assuredly need to be ported to more facile and lower-cost assay formats. Such formats would likely target a prioritized subset of biomarkers using high-specificity affinity reagents. Regarding limitations of our study, while we carefully considered our patient recruitment criteria, our cohort was not necessarily completely representative of the comprehensive endpoint population. Additionally, because machine learning classifier performance for our proteomic signatures approached but did not exceed that of a current standard-of-care benchmark for prostate cancer detection, biomarker candidates identified in this study would likely need to be combined with those from other similar discovery-mode efforts to meet performance thresholds sufficient for new clinical test development. Despite the overall challenge of developing new tests for improved prostate cancer diagnosis, our study contributes significant value to this overall effort in using differentiating and powerful proteomic technologies to uncover new marker candidates in a scaled cohort of patient serum. Differentially abundant biomarker candidates identified here provide critical inputs for the development of new clinical tests aimed towards more effective prostate cancer detection with reduced frequency of unnecessary prostate biopsy.

## Figures and Tables

**Table 1:** Description of SABOR and Finasteride cohorts. Fields where data was not available are labeled as NA.

	All Patients		PSA 4–10 ng/mL	
	SABOR	Finasteride	SABOR	Finasteride
	No. 657	No. 265	No. 212	No. 217
Age at biopsy				
Median (IQR)	64.0 (58.0–70.0)	65.0 (61.0–68.0)	67.0 (61.0–73.0)	65.0 (61.0–68.0)
Missing	1 (0.2%)	0 (0%)	1 (0.5%)	0 (0%)
Race/ethnicity				
Other	6 (0.9%)	3 (1.1%)	2 (0.9%)	2 (0.9%)
Non-Hispanic Black	80 (12.2%)	33 (12.5%)	24 (11.3%)	28 (12.9%)
Non-Hispanic White	401 (61.0%)	132 (49.8%)	123 (58.0%)	111 (51.2%)
Hispanic	170 (25.9%)	97 (36.6%)	63 (29.7%)	76 (35.0%)
Year of biopsy				
(1999, 2004]	218 (33.2%)	0 (0.0%)	69 (32.5%)	0 (0.0%)
(2004, 2009]	347 (52.8%)	0 (0.0%)	103 (48.6%)	0 (0.0%)
(2009, 2014]	67 (10.2%)	208 (78.5%)	31 (14.6%)	166 (76.5%)
(2014, 2019]	25 (3.8%)	57 (21.5%)	9 (4.2%)	51 (23.5%)
Family history of prostate cancer				
No	368 (56.0%)	218 (82.3%)	138 (65.1%)	178 (82.0%)
Yes	289 (44.0%)	47 (17.7%)	74 (34.9%)	39 (18.0%)
Prior biopsy	218 (33.2%)	42 (15.8%)	90 (42.5%)	36 (16.6%)
DRE results				
Abnormal	169 (25.7%)	20 (7.5%)	41 (19.3%)	11 (5.1%)
Normal	380 (57.8%)	243 (91.7%)	127 (59.9%)	204 (94.0%)
No DRE/unknown result	108 (16.4%)	2 (0.8%)	44 (20.8%)	2 (0.9%)
PSA (ng/mL)				
Median (IQR)	3.3 (1.6–5.0)	5.2 (4.3–6.5)	5.3 (4.7–6.8)	5.4 (4.7–6.7)
Missing	2 (0.3%)	0 (0%)	0 (0%)	0 (0%)
PCPT risk score—prostate				
Median (IQR)	22.0 (17.0–29.0)	28.0 (25.0–32.0)	29.0 (24.0–33.0)	28.0 (26.0–32.0)
Missing	5 (0.8%)	0 (0%)	3 (1.4%)	0 (0%)
PCPT risk score—GS > 6 cancer				
Median (IQR)	5.0 (3.0–9.0)	8.0 (7.0–11.0)	9.0 (7.0–13.0)	8.0 (7.0–11.0)
Missing	5 (0.8%)	0 (0%)	3 (1.4%)	0 (0%)
Number of biopsy cores				
<12	144 (21.9%)	9 (3.4%)	46 (21.7%)	8 (3.7%)
12	413 (62.9%)	248 (93.6%)	132 (62.3%)	204 (94.0%)
>12	99 (15.1%)	8 (3.0%)	34 (16.0%)	5 (2.3%)
Missing	1 (0.2%)	0 (0.0%)	0 (0.0%)	0 (0.0%)
Cancer status				
No cancer detected (negative biopsy + no dx within 18 months)	349 (53.1%)	132 (49.8%)	97 (45.8%)	101 (46.5%)
Cancer detected on initial biopsy	308 (46.9%)	130 (49.1%)	115 (54.2%)	114 (52.5%)
Negative initial biopsy but cancer dx within 18 months	NA	3 (1.1%)	NA	2 (0.9%)
Biopsy Gleason score				
GS ≤ 6	188 (28.6%)	62 (23.4%)	61 (28.8%)	55 (25.3%)
GS ≥ 7	119 (18.1%)	68 (25.7%)	53 (25.0%)	59 (27.2%)
Missing	1 (0.2%)	0 (0.0%)	1 (0.5%)	0 (0.0%)
Prostatectomy Gleason Grade (within 18 months of serum collection)				
GG 1 (GS ≤ 6)	NA	4 (1.5%)	NA	2 (0.9%)
GG ≥ 2 (GS ≥ 7)	NA	16 (6.0%)	NA	15 (6.9%)
Summary Gleason Grade Group				
GG 1 (GS ≤ 6)	189 (28.8%)	60 (22.6%)	62 (29.2%)	53 (24.4%)
GG ≥ 2 (GS ≥ 7)	119 (18.1%)	73 (27.5%)	53 (25.0%)	63 (29.0%)

**Table 2:** Classifier performance for the protein multimarker (MM) signature and PCPT risk score for the validation methods employed in the study. Performance values represent the point estimate for the respective metric, with the 95% confidence interval shown in brackets.

	Subjects	Definition	Metric	MM Performance	PCPT Performance
Nested Cross-Validation in Training Set	PSA 4–10	1	AUC	0.64 [0.58, 0.70]	0.60 [0.54, 0.67]
	PSA 4–10	1	NPV	0.62 [0.56, 0.67]	0.56 [0.48, 0.65]
	PSA 4–10	1	PPV	0.65 [0.59, 0.71]	0.62 [0.53, 0.71]
	PSA 4–10	2	AUC	0.62 [0.56, 0.68]	0.60 [0.52, 0.67]
	PSA 4–10	2	NPV	0.74 [0.67, 0.81]	0.79 [0.72, 0.85]
	PSA 4–10	2	PPV	0.34 [0.30, 0.37]	0.35 [0.27, 0.45]
Validation in Withheld Test Set	PSA 4–10	1	AUC	0.48 [0.39, 0.58]	0.70 [0.61, 0.79]
	PSA 4–10	1	NPV	0.41 [0.31, 0.53]	0.67 [0.55, 0.79]
	PSA 4–10	1	PPV	0.60 [0.49, 0.71]	0.68 [0.57, 0.82]
	PSA 4–10	2	AUC	0.51 [0.40, 0.62]	0.68 [0.58, 0.78]
	PSA 4–10	2	NPV	0.75 [0.66, 0.86]	0.86 [0.78, 0.92]
	PSA 4–10	2	PPV	0.25 [0.17, 0.37]	0.39 [0.26, 0.56]
Bootstrap Bias-Corrected Cross-Validation	PSA 4–10	1	AUC	0.59 [0.52, 0.65]	0.63 [0.56, 0.70]
	PSA 4–10	1	NPV	0.54 [0.45, 0.62]	0.59 [0.49, 0.69]
	PSA 4–10	1	PPV	0.61 [0.52, 0.70]	0.64 [0.55, 0.74]
	PSA 4–10	2	AUC	0.53 [0.48, 0.58]	0.62 [0.54, 0.69]
	PSA 4–10	2	NPV	0.74 [0.66, 0.81]	0.81 [0.74, 0.87]
	PSA 4–10	2	PPV	0.28 [0.21, 0.35]	0.36 [0.26, 0.47]
	All	1	AUC	0.61 [0.54, 0.67]	0.64 [0.57, 0.71]
	All	1	NPV	0.56 [0.47, 0.65]	0.60 [0.50, 0.70]
	All	1	PPV	0.62 [0.53, 0.70]	0.64 [0.55, 0.74]
	All	2	AUC	0.52 [0.49, 0.59]	0.62 [0.55, 0.70]
	All	2	NPV	0.71 [0.64, 0.79]	0.81 [0.74, 0.88]
	All	2	PPV	0.26 [0.20, 0.33]	0.36 [0.27, 0.47]

## Data Availability

All raw MS data were deposited at MassIVE on July 2024. The data is publicly accessible at ftp://massive.ucsd.edu/v08/MSV000095357/.

## Supplementary Materials

The supporting information can be downloaded at: https://www.mdpi.com/article/10.3390/ijms25158010/s1.
