# Supplementary material for: A Scaled Proteomic Discovery Study for Prostate Cancer Diagnostic Markers Using ProteographTM and Trapped Ion Mobility Mass Spectrometry
Source: Int J Mol Sci. 2024 Jul 23;25(15):8010. doi: 10.3390/ijms25158010 (PMC11311733; doi:10.3390/ijms25158010)
Supplement: Supplementary file 1 [file ijms-25-08010-s001.zip › Changetal_manuscript_submission_20240611_supplemental_figures.pdf]

SUPPLEMENTARY FIGURES

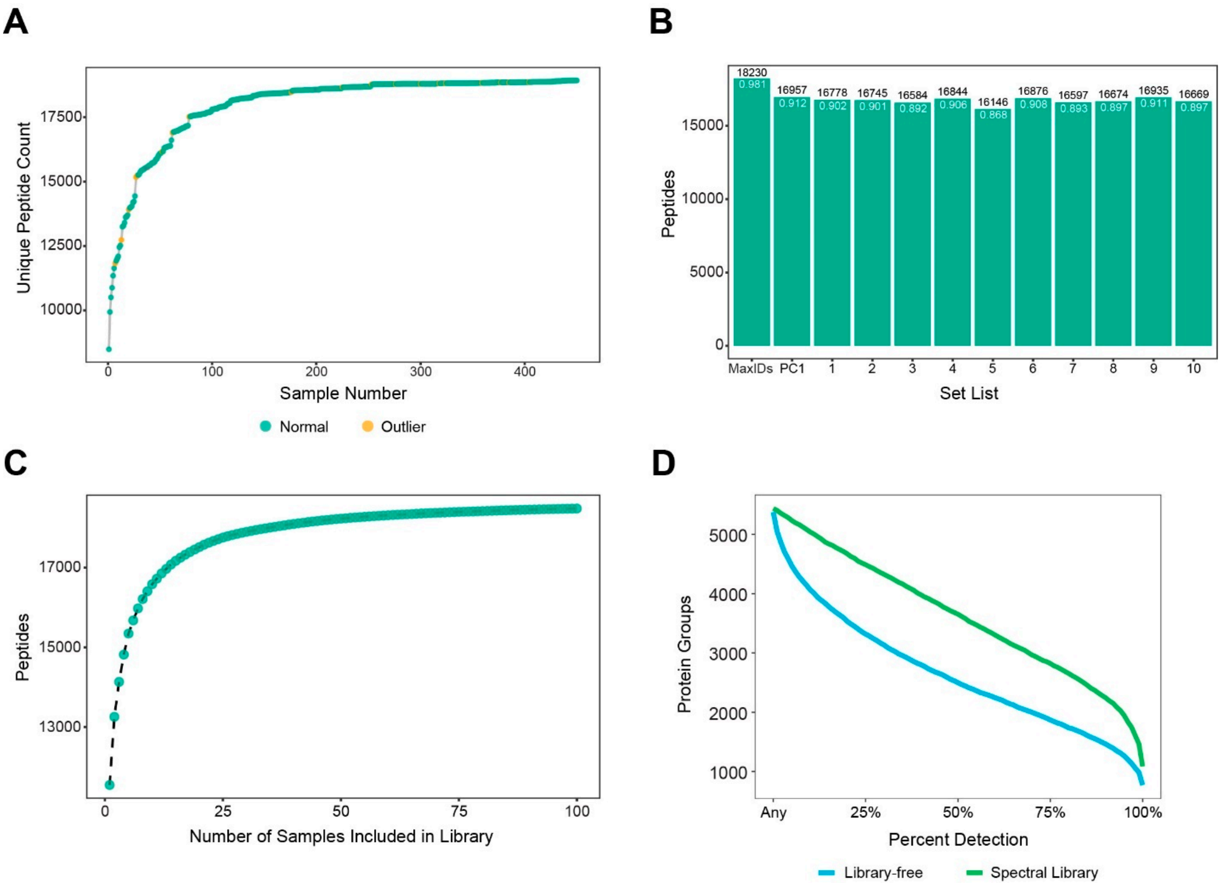

Figure S1. Spectral library sample selection methodology. (**A**) Aggregate unique peptides for each successive sample added to the total number of samples to assess when additional samples would contribute minimally to the total unique proteomic diversity in the sample set. Normal vs Outlier samples are described in detail in the Methods. (**B**) The number of peptides comprising the samples selected from three different selection strategies. PC1 used the first principal component scores from PCA to select samples evenly dispersed across the PC1 range. Samples were also randomly selected, which are represented by 1-10. MaxIDs, described further in the Methods, iteratively selects samples with the maximum number of peptide identifications from the remaining pool of samples. Total peptides for each selection are shown above each bar and the fraction of these peptides from the total peptides in all samples is below. (**C**) MaxIDs modeling the number of peptides identified from the samples selected for a given set size. (**D**) Raw dia-PASEF data for the training set was searched in DIA-NN using the study-specific spectral library or in library-free mode. Protein group identifications at a given percent detection threshold across all samples are shown between the two search methods.

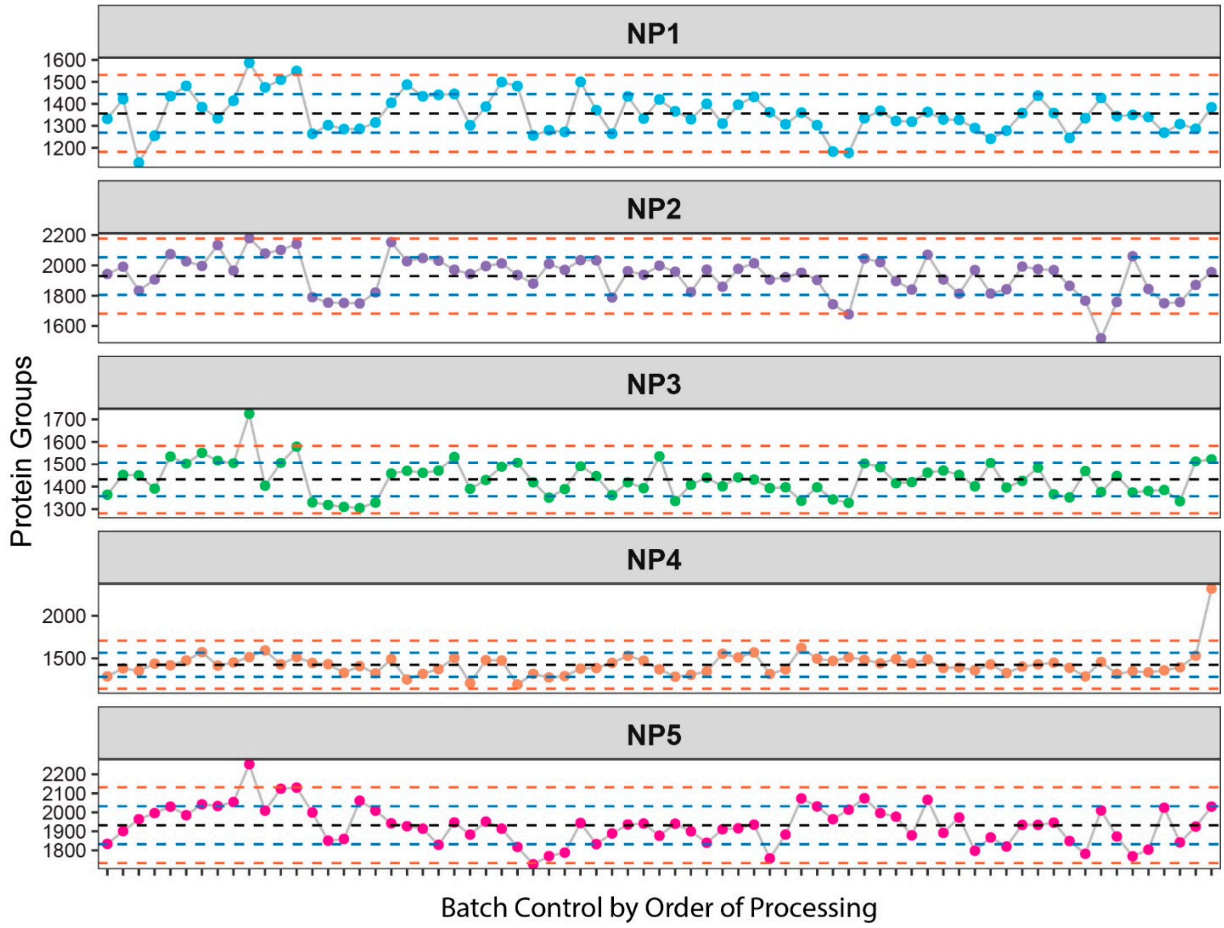

Figure S2. Sample processing and LC-MS acquisition performance monitoring. Each plate of samples was prepared with a common process control PC3 to simultaneously monitor the fidelity of sample processing with Proteograph^{TM} and to assess LC-MS performance from batch-to-batch. The plot shows protein group identifications for PC3 from each batch in the order they were prepared with the black dashed line representing mean protein groups, the blue dashed line ± 1 × sd, and orange dashed line ± 2 × sd. Control samples that fell below the 2 × sd line triggered an assessment of one or more modules of the workflow.

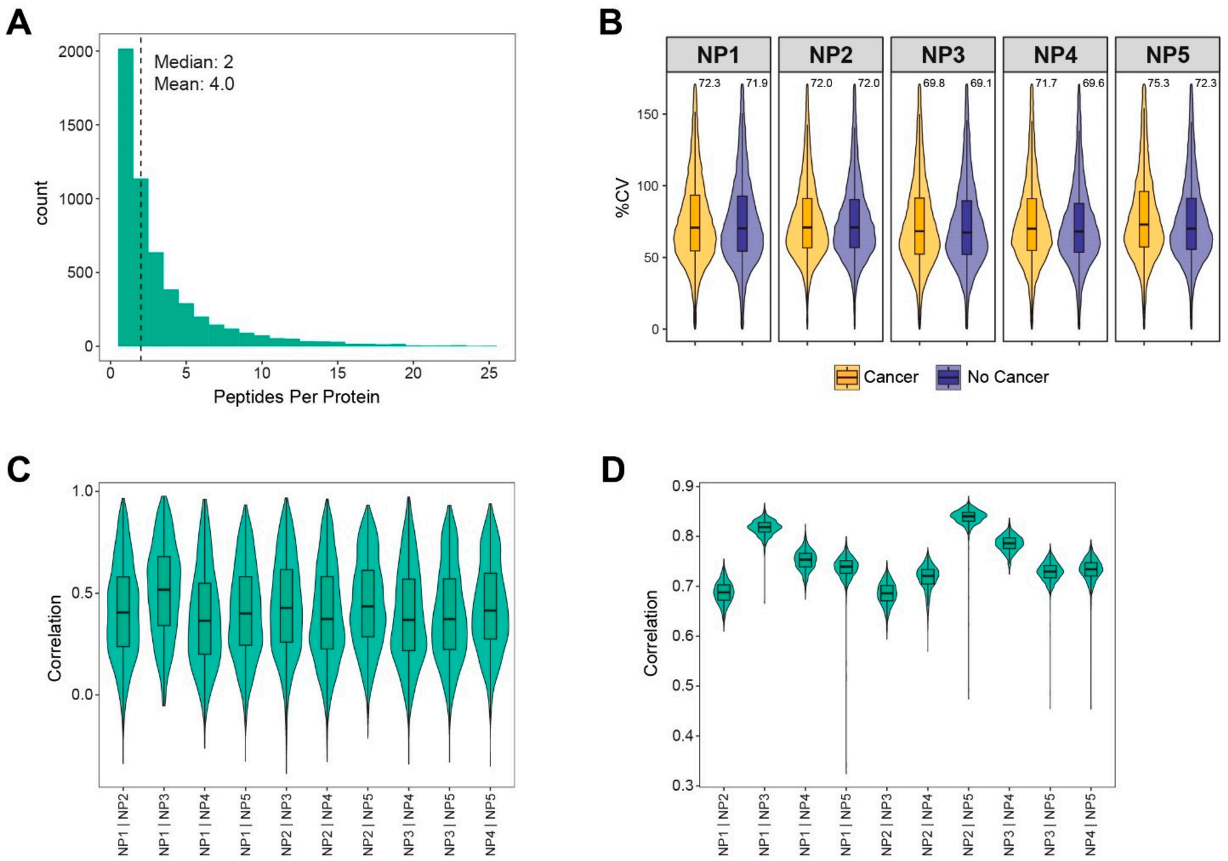

Figure S3. Additional characterization of proteomic performance and dynamics of protein group measurements across nanoparticles. (**A**) Histogram of peptides identified per protein identification with the median number of peptides indicated by the dashed line. (**B**) Percent coefficient of variation distribution for peptide intensities per nanoparticle are shown for both case and control with the inner boxplots indicating the 25, 50, and 75th percentiles, respectively, and whiskers representing ±1.5 x IQR. Outliers are not included. Outer violins encompass data within the plotting range displayed. (**C**) Distribution of Pearson's pairwise correlation coefficients for a given protein group MaxLFQ intensities between the indicated nanoparticles across all samples (intra-protein, across samples where each point in the distribution represents the correlation for a protein group between one NP to another for all subjects). Boxplots and violin plots are representative of the same summary statistics as indicated above except outliers are included. (**D**) Distribution of Pearson's pairwise correlation coefficients for a given sample and all matched protein group intensities in one nanoparticle enrichment against another (intra-samples across proteins where each point in the distribution is the correlation of a subject between one NP to another for all protein groups). Parameters displayed in boxplots and violin plots are the same as shown above.

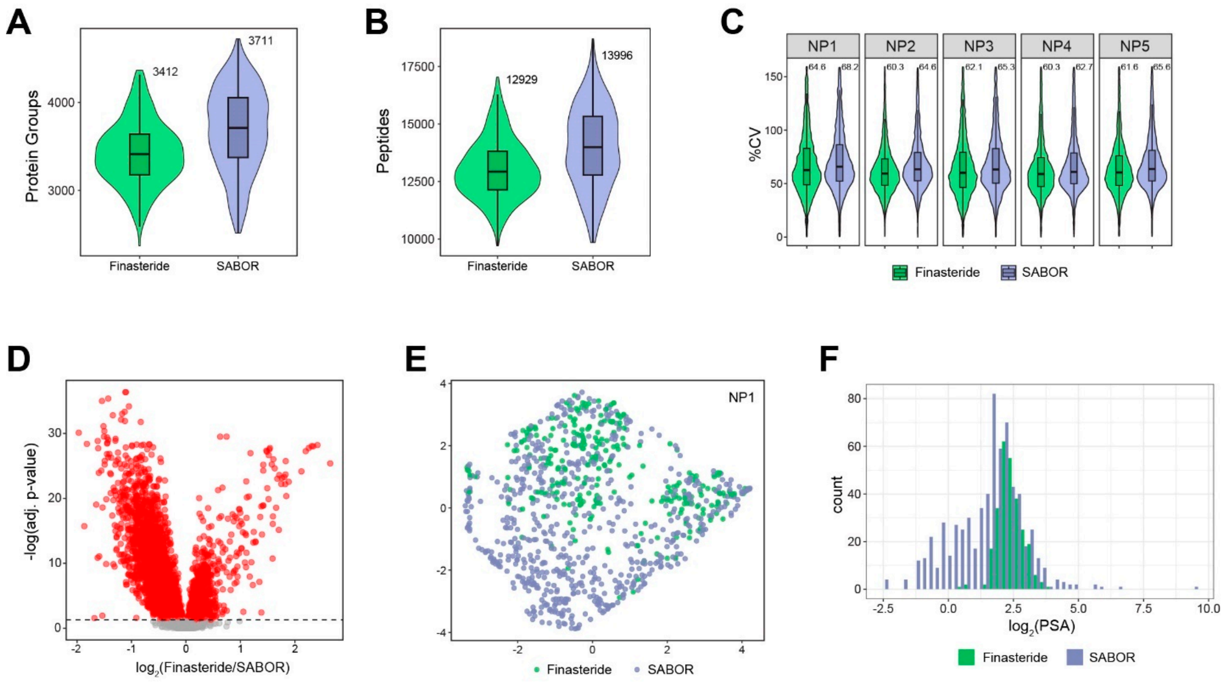

Figure S4. Proteomic differences between subjects in the Finasteride and SABOR cohorts. (**A**) Distribution of protein group identifications and (**B**) peptides between samples in the two cohorts. Median proteins and peptides are noted for each group. Boxplots and violins represent the same statistics as indicated in the figures above. (**C**) Distribution of percent coefficient of variation (%CV) calculated from MaxLFQ protein intensities for each cohort per nanoparticle. Median %CV for each group is displayed. (**D**) Volcano plot showing differential protein-NP expression between subjects in Finasteride vs SABOR. Statistical significance was determined by a Wilcox test on proteins-NP features identified in ≥ 40% of samples. Highlighted protein-NP features have an adjusted p-value < 0.05. (**E**) UMAP from protein intensities in NP1 with subjects colored by cohort. Only protein groups detected in ≥ 40% of samples were used as input. PCA scores were calculated from these features and the first 25 principal components then used in UMAP. (**F**) Distribution of log_2-transformed PSA measurements from each subject separated by cohort.

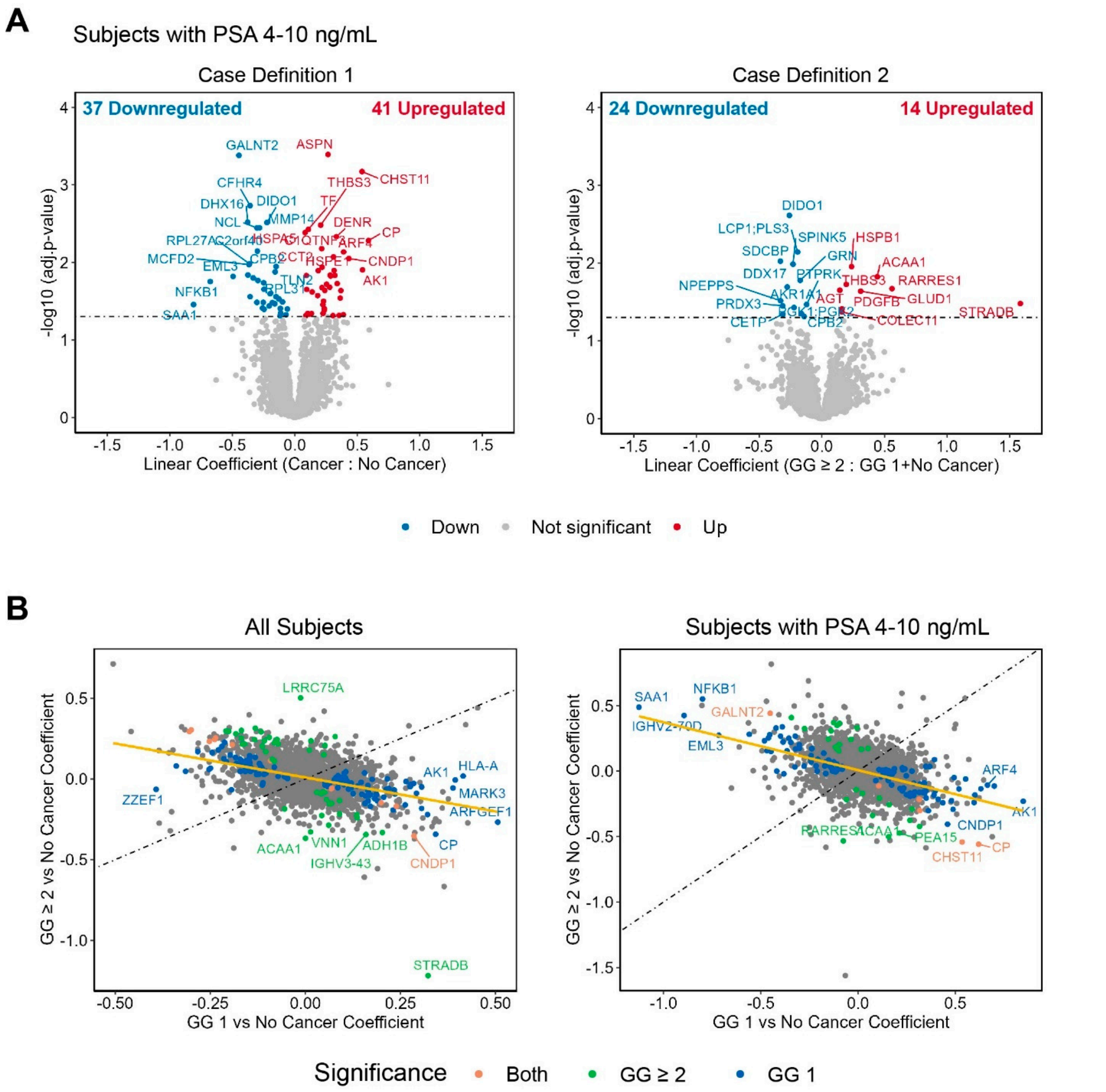

Figure S5. Differential expression analysis in patients with PSA 4-10 ng/mL and a comparison between abundance differences between lower-grade GG 1 and GG ≥ 2 vs no cancer controls. (**A**) Volcano plot showing differential protein abundance as determined with a linear mixed model for case definition 1 (left) and case definition 2 (right). Proteins with an adjusted p-value < 0.05 are highlighted with log2 fold changes in protein intensity indicated by the linear coefficient. (**B**) Scatter plot of GG 1 vs no cancer log2 differential protein abundance as compared to that in GG ≥ 2 vs no cancer. Proteins with statistically significant differential expression (adjusted p-value < 0.05) in only the GG 1 comparison are indicated in blue, only in GG ≥ 2 in green, and when both passed the FDR cutoff in orange. The dashed line, y = x, represents R = 1 correlation between the two log2 fold changes in protein abundance and the resulting linear regression from the scatter plot by the yellow line.

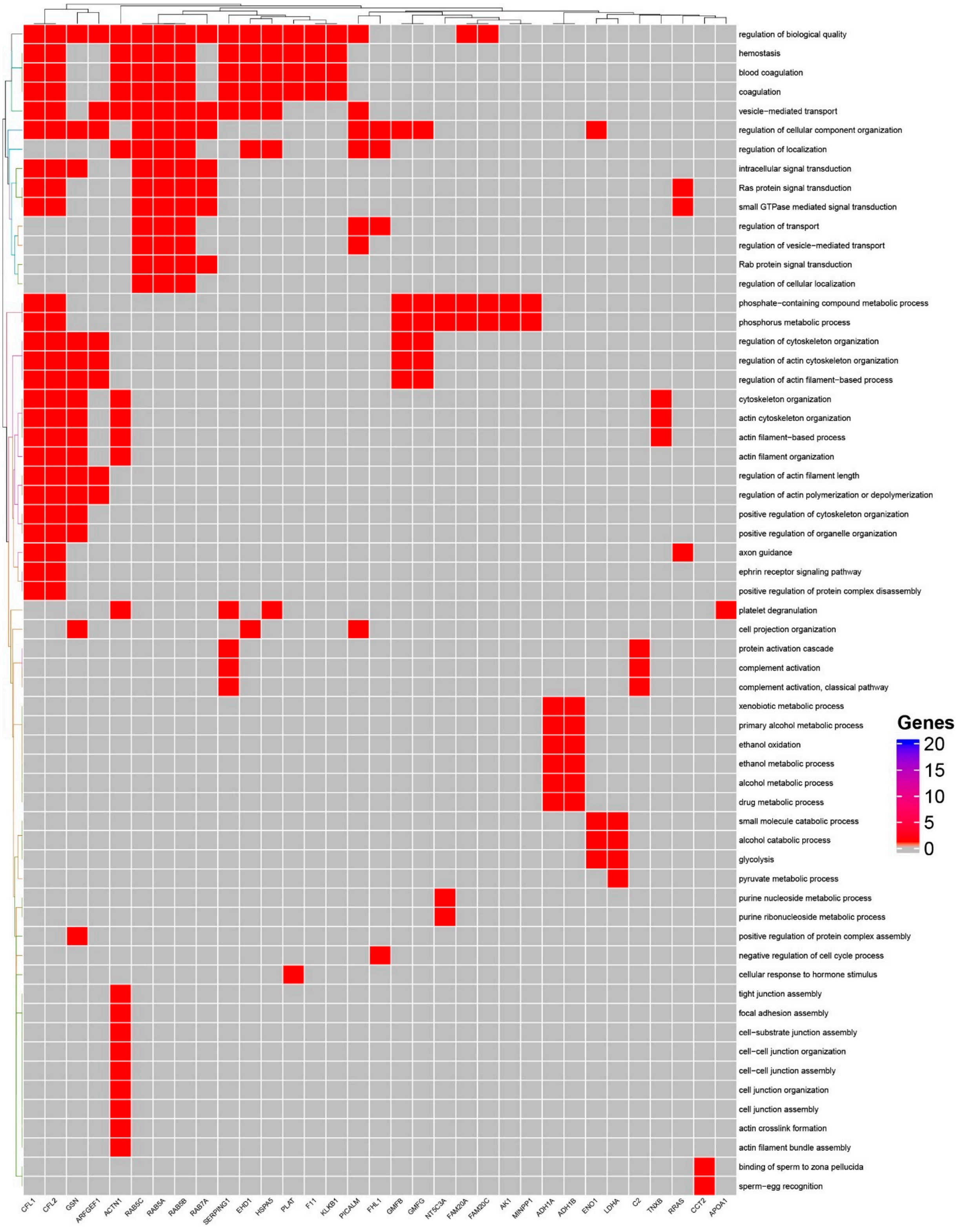

Figure S6. Clustering of upregulated gene ontology (GO) terms from the 1D annotation enrichment on the differentially abundant proteins in case definition 1.

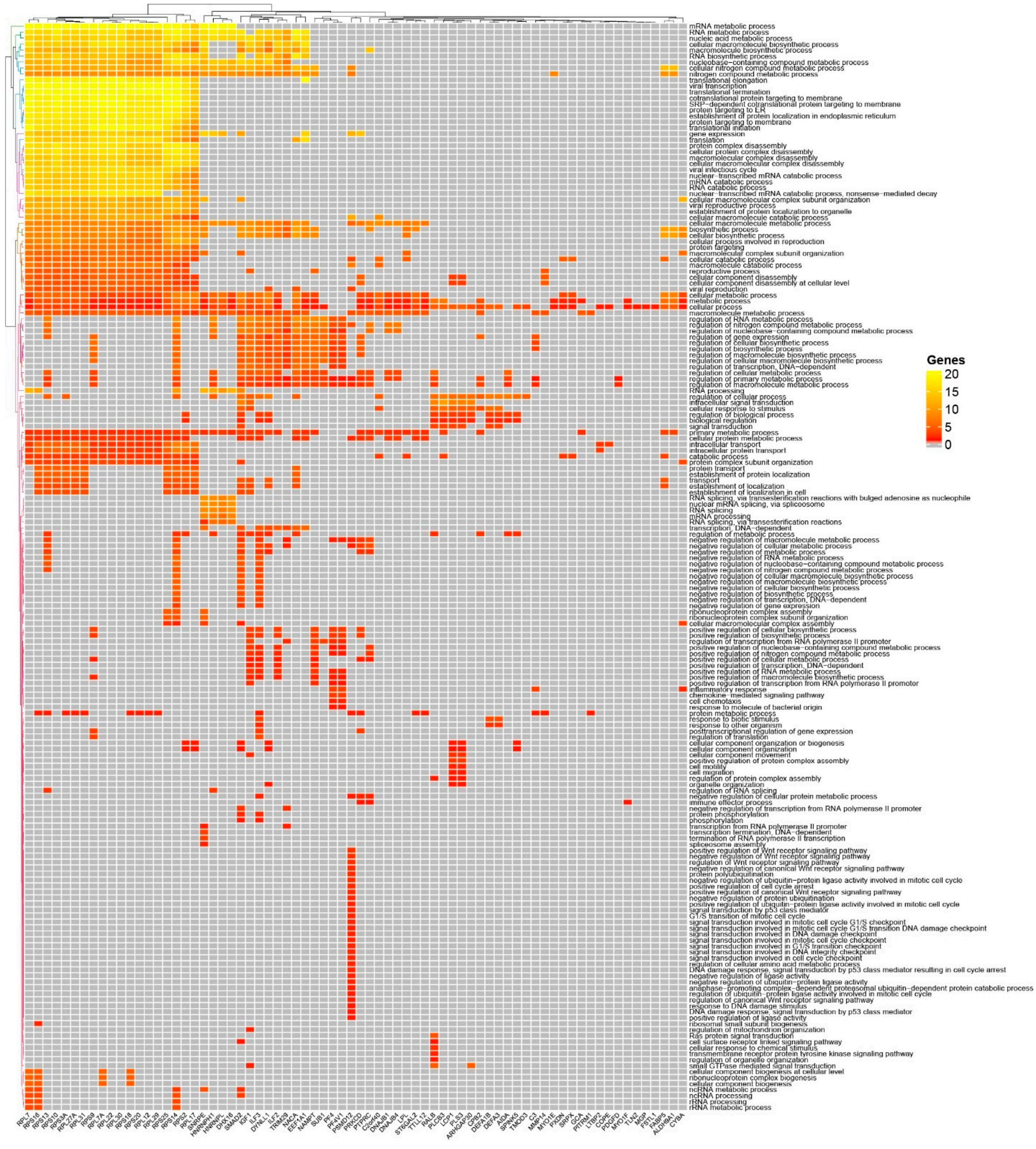

Figure S7. Clustering of downregulated gene ontology (GO) terms from the 1D annotation enrichment on the differentially abundant proteins in case definition 1.

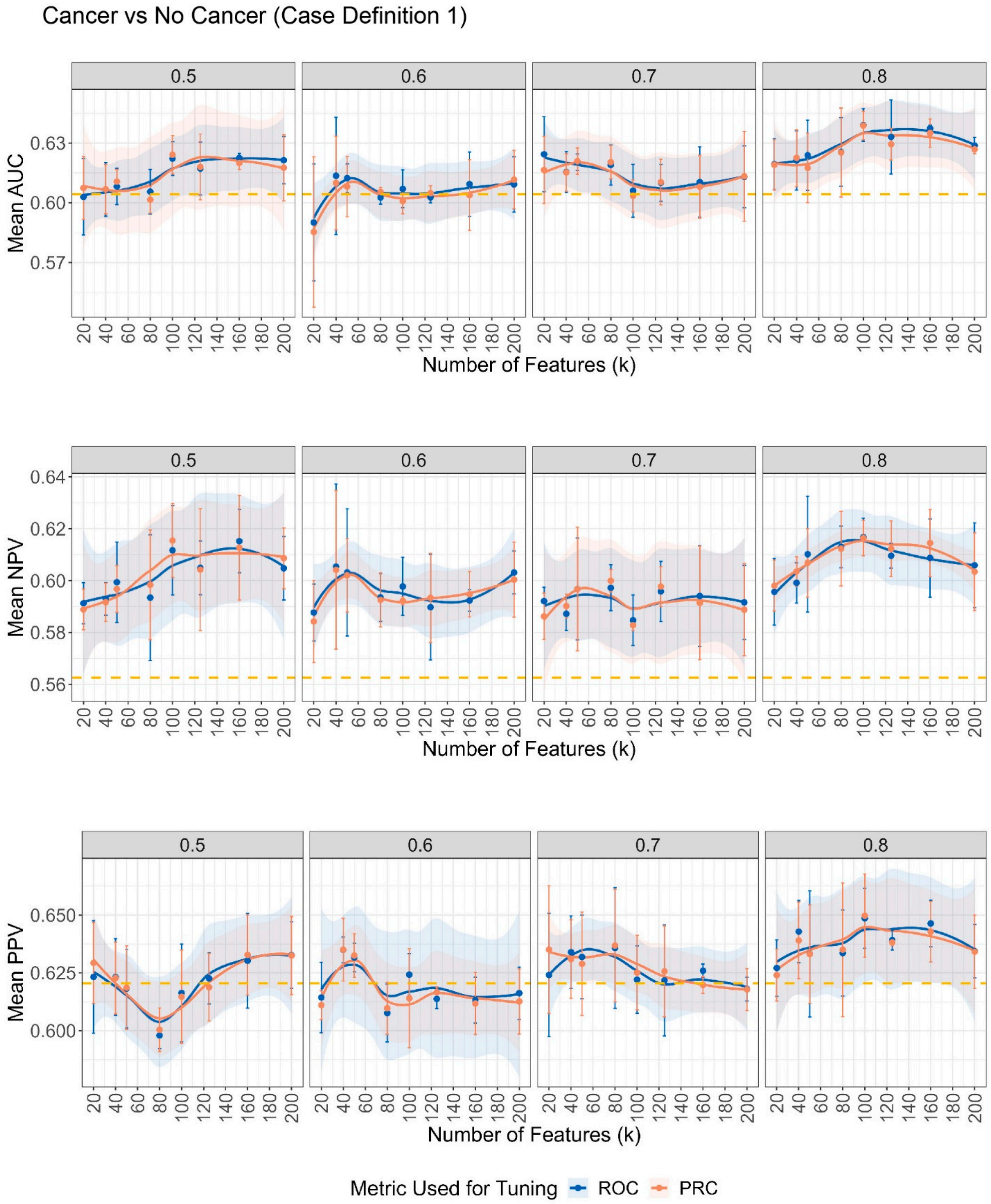

Figure S8. Exploration and optimization of feature selection hyperparameters of the ML classifier model comparing cancer vs no cancer. Only subjects with PSA 4-10 ng/mL and from the training set of the study were used. The number of features (k) used in the random forest model, the correlation cutoff to remove correlated features (displayed in the grey boxes above

each plot), and the performance metric, area under ROC or PRC, used to tune the model were investigated and evaluated according to mean AUC, NPV, and PPV across 10-fold cross validation and three separate random seeds. The performance of the PCPT risk scores in the training set is displayed as a dashed yellow line for the respective metrics.

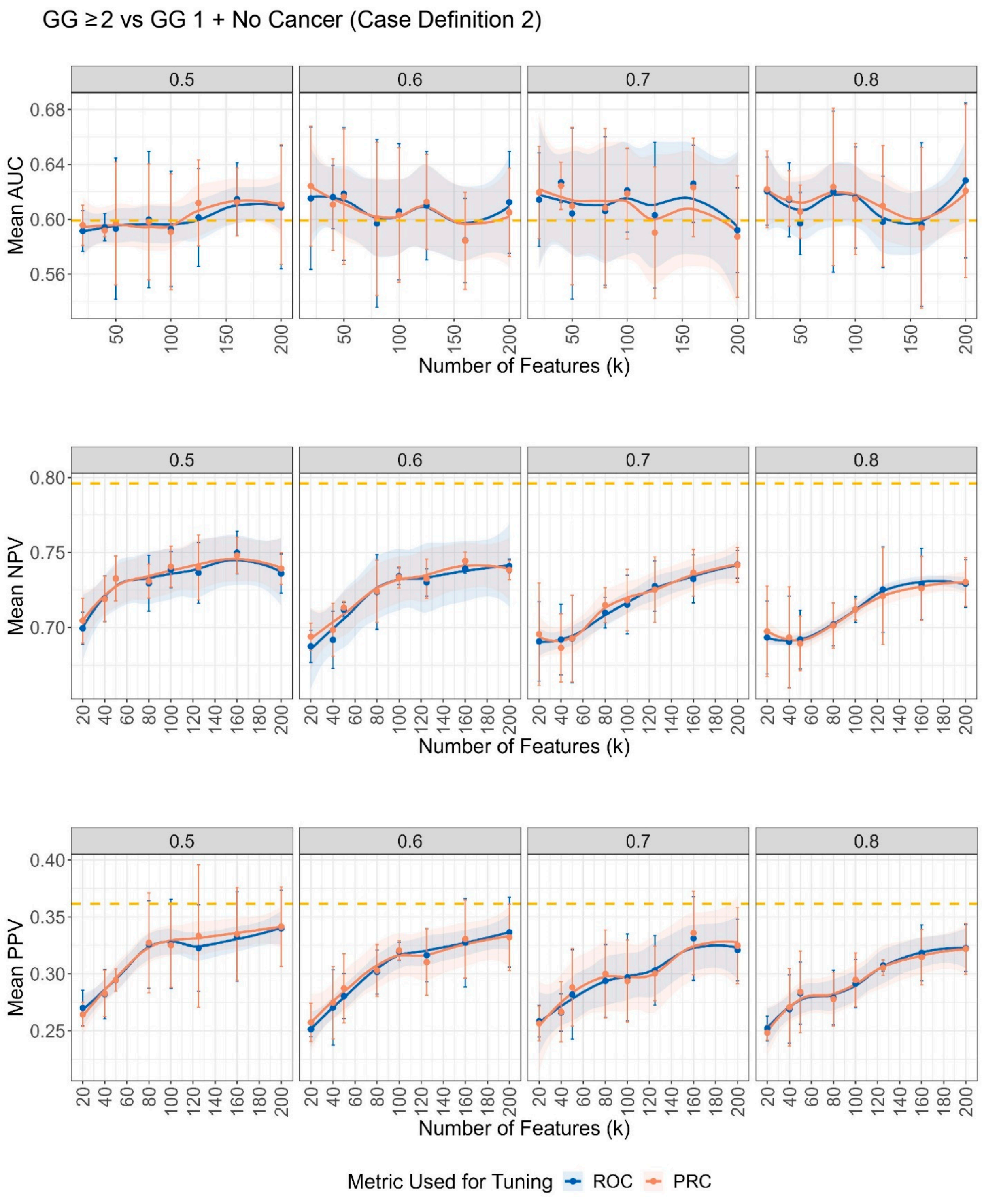

Figure S9. Exploration and optimization of feature selection hyperparameters of the ML classifier model comparing GG ≥ 2 cancer vs GG 1 cancer and no cancer. Only subjects with PSA 4-10 ng/mL and from the training set of the study were used. The number of features (k) used in the random forest model, the correlation cutoff to remove correlated features (displayed

in the grey boxes above each plot), and the performance metric, area under ROC or PRC, used to tune the model were investigated and evaluated according to mean AUC, NPV, and PPV across 10-fold cross validation and three separate random seeds. The performance of the PCPT risk scores in the training set is displayed as a dashed yellow line for the respective metrics.

Cancer vs No Cancer (Case Definition 1)

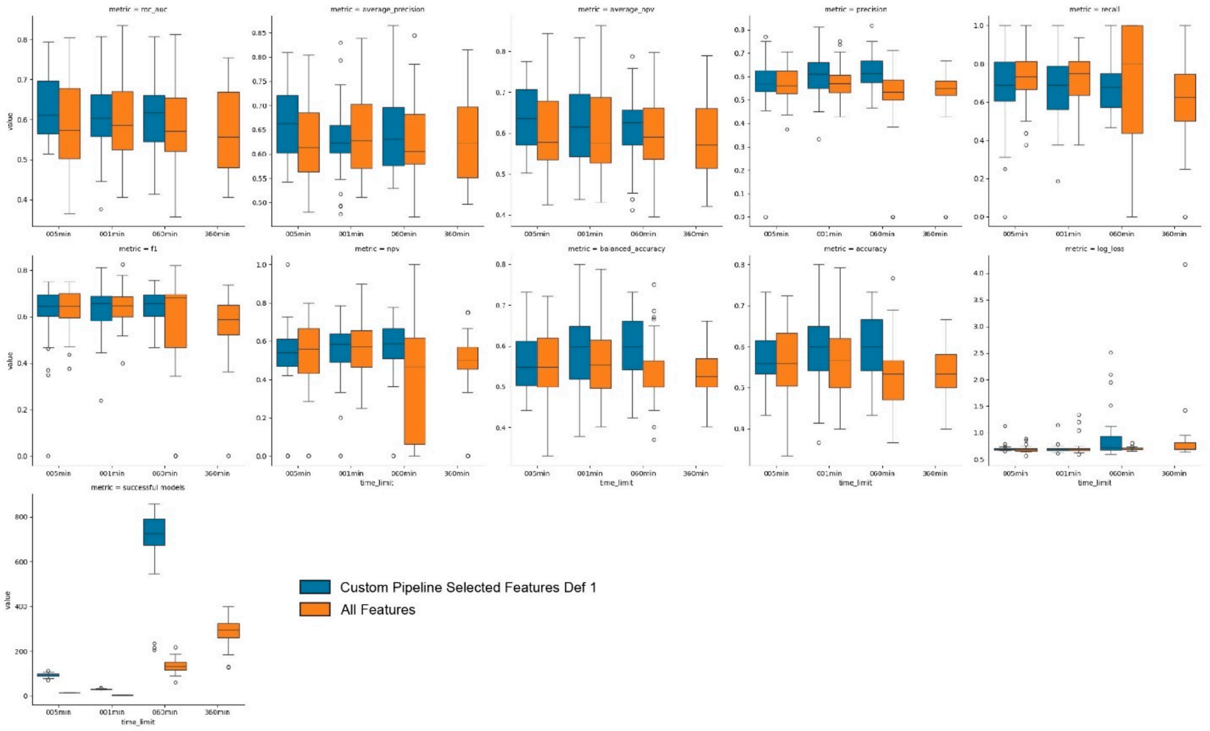

GG ≥ 2 vs GG 1 + No Cancer (Case Definition 2)

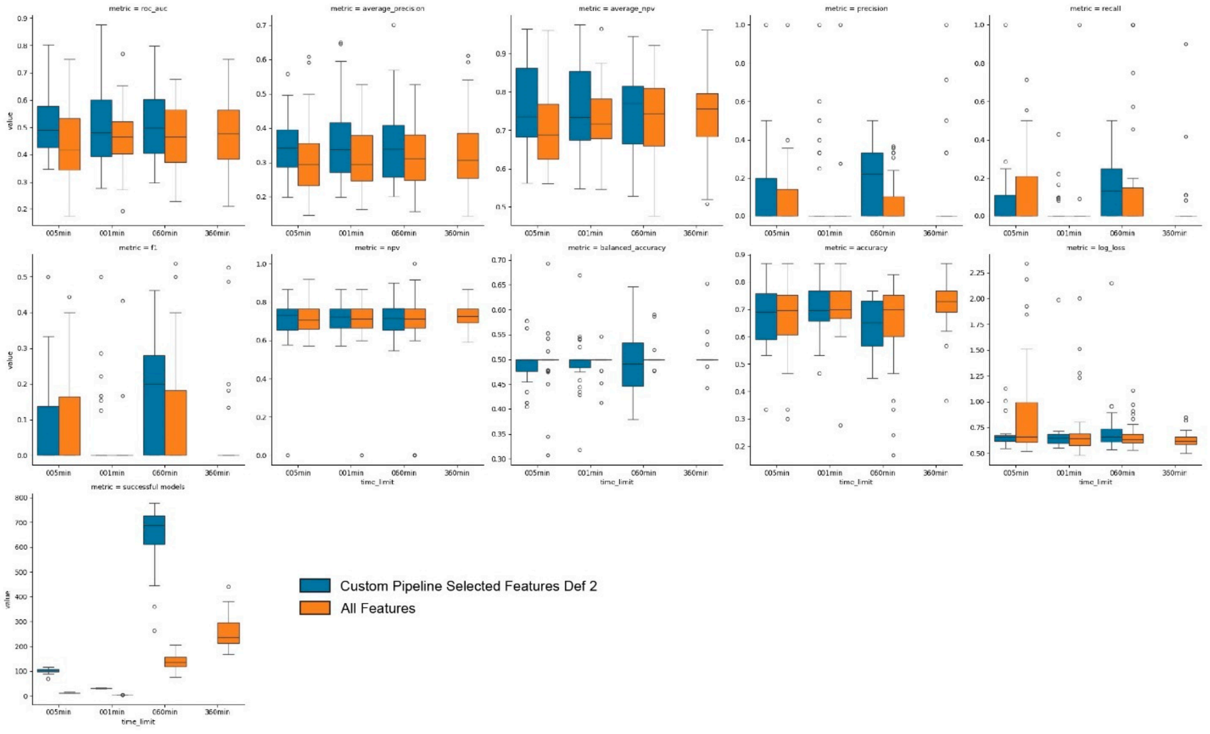

Figure S10. Distributions of summary metrics from AutoML in the training set data for case definition 1 and 2. AutoML used only the protein features from the custom pipeline, acting as a replacement for random forest as the classifier in the custom pipeline, or utilized all features.

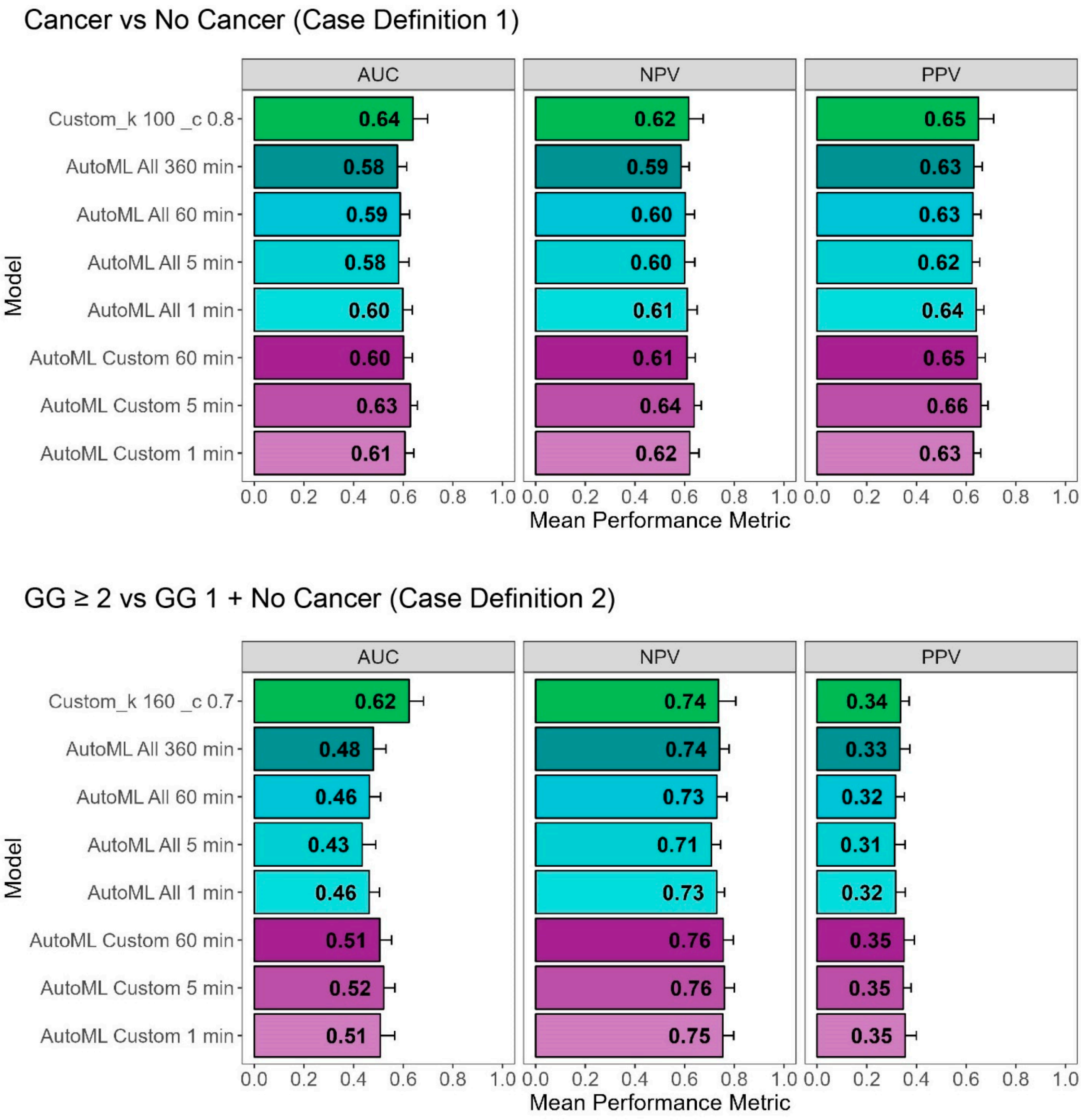

Figure S11. Classifier performance of the custom pipeline for subjects with PSA 4-10 ng/mL in the training set as compared to AutoML. In the assessment, AutoML replaced random forest as the classifier for the custom pipeline or was allowed full access to all protein features in the dataset to train a classifier model. Matched subject allocations between training and test splits in a nested cross-validation scheme across three random seeds were used for AutoML and the

custom pipeline. AutoML was allocated different time allotments for the two different feature configurations. Mean values for AUC, NPV, and PPV are displayed in the respective plots for the different models with 95% confidence intervals indicated by the error bars.

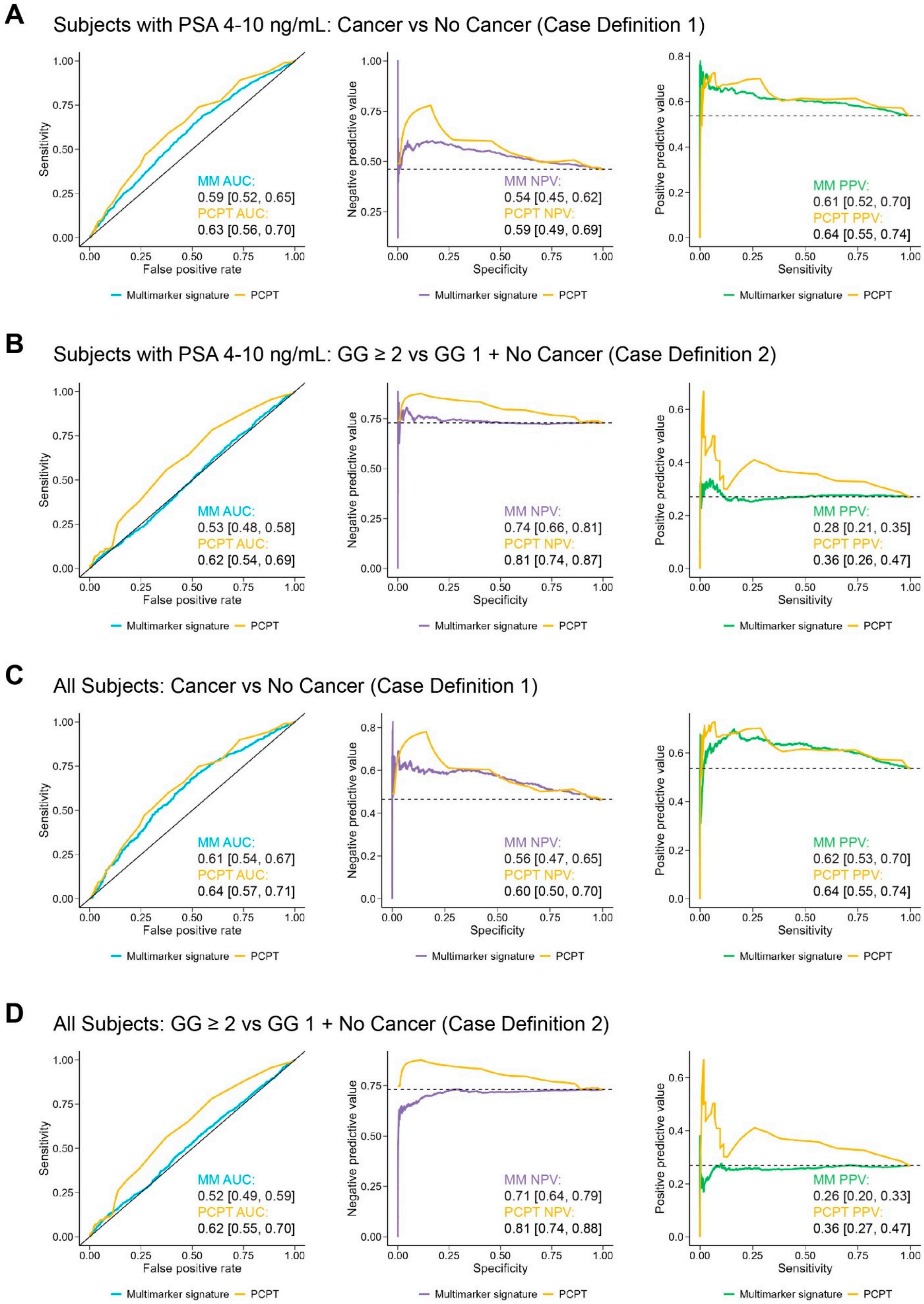
A
Subjects with PSA 4-10 ng/mL: Cancer vs No Cancer (Case Definition 1)
Sensitivity
False positive rate
MM AUC:
0.59 [0.52, 0.65]
PCPT AUC:
0.63 [0.56, 0.70]
Negative predictive value
Specificity
MM NPV:
0.54 [0.45, 0.62]
PCPT NPV:
0.59 [0.49, 0.69]
Positive predictive value
Sensitivity
MM PPV:
0.61 [0.52, 0.70]
PCPT PPV:
0.64 [0.55, 0.74]
Multimarker signature
PCPT
B
Subjects with PSA 4-10 ng/mL: GG ≥ 2 vs GG 1 + No Cancer (Case Definition 2)
MM AUC:
0.53 [0.48, 0.58]
PCPT AUC:
0.62 [0.54, 0.69]
MM NPV:
0.74 [0.66, 0.81]
PCPT NPV:
0.81 [0.74, 0.87]
MM PPV:
0.28 [0.21, 0.35]
PCPT PPV:
0.36 [0.26, 0.47]
C
All Subjects: Cancer vs No Cancer (Case Definition 1)
MM AUC:
0.61 [0.54, 0.67]
PCPT AUC:
0.64 [0.57, 0.71]
MM NPV:
0.56 [0.47, 0.65]
PCPT NPV:
0.60 [0.50, 0.70]
MM PPV:
0.62 [0.53, 0.70]
PCPT PPV:
0.64 [0.55, 0.74]
D
All Subjects: GG ≥ 2 vs GG 1 + No Cancer (Case Definition 2)
MM AUC:
0.52 [0.49, 0.59]
PCPT AUC:
0.62 [0.55, 0.70]
MM NPV:
0.71 [0.64, 0.79]
PCPT NPV:
0.81 [0.74, 0.88]
MM PPV:
0.26 [0.20, 0.33]
PCPT PPV:
0.36 [0.27, 0.47]

Figure S12. Summary of classifier performance of the multimarker signature (MM). ROC, NPV-Specificity, and Precision-Recall curves are shown for the protein-level data and the PCPT risk score in subjects with PSA 4-10 ng/mL for case definition 1 (**A**) and case definition 2 (**B**), and for all subjects in the study (**C**) and (**D**). Average AUC, NPV, and PPV for the protein signature and the PCPT risk score are displayed for each respective plot with the 95% confidence interval indicated in brackets.

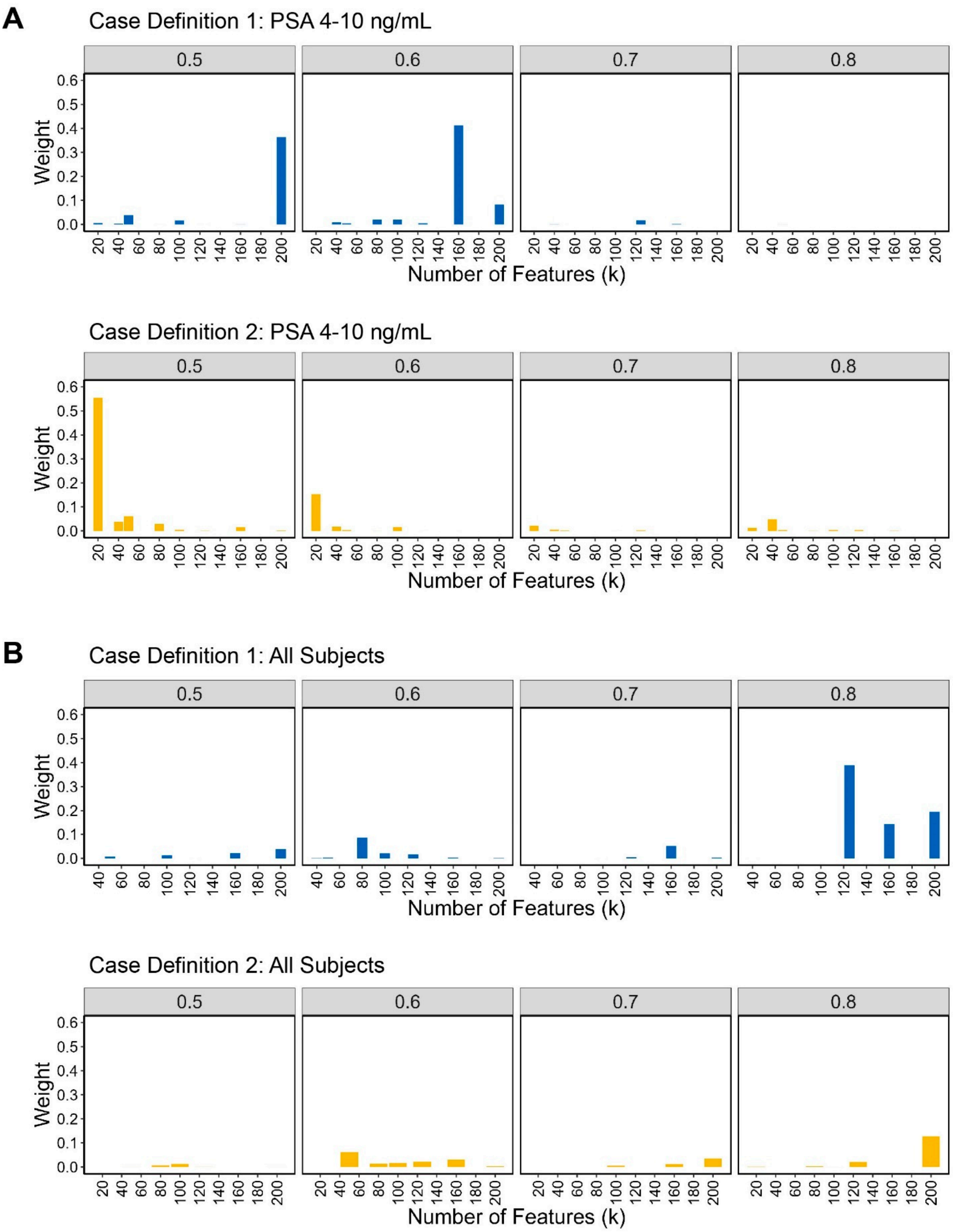

Figure S13. Weighted contribution for each model selected in the bootstrap bias-corrected cross-validation for subjects restricted to a PSA 4-10 ng/mL (**A**) and all subjects (**B**). Model

configuration is indicated by the number of protein features (k) used in the model, the correlation cutoff to remove correlated features (stripped text in grey), and the performance metric used to tune the hyperparameters (AUC for case definition 1 and area under PRC for case definition 2).

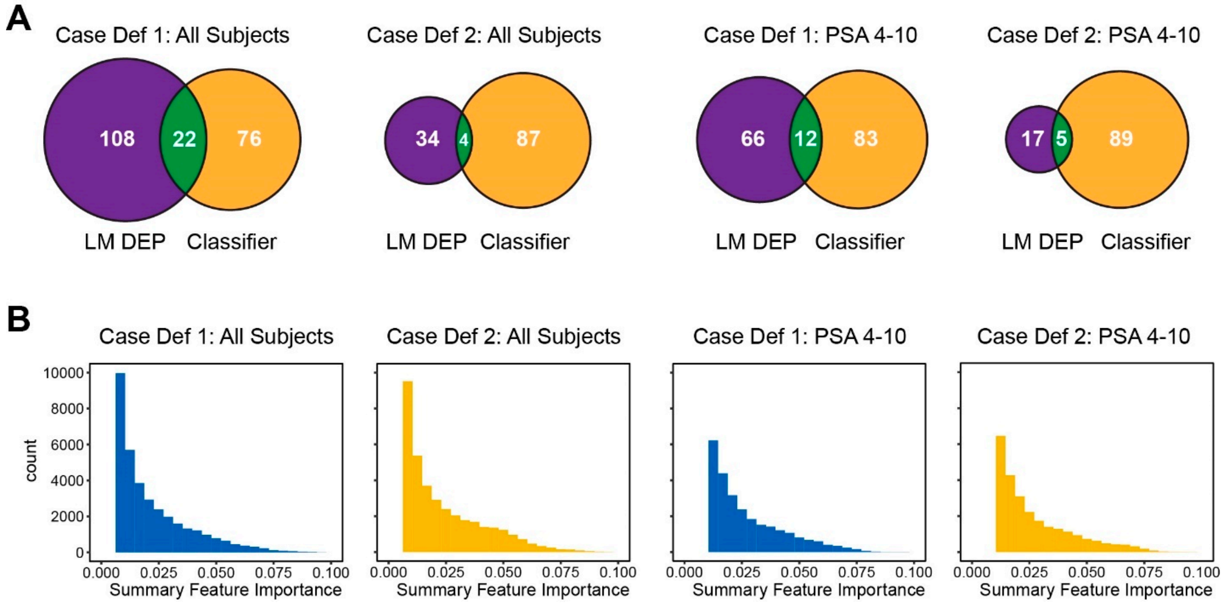

Figure S14. Analysis summary of the top 100 features selected from the bootstrap bias-corrected cross-validation (BBC-CV). (**A**) Overlap of protein groups between the differentially expressed proteins from the linear mixed model (LM DEP) and those corresponding to the top 100 features from the protein multimarker classifier as summarized from the weighted feature importance score from the BBC-CV. (**B**) Distribution of feature importance scores for all protein features appearing across selected models in the BBC-CV.
